# Estimating the economic burden of diabetes in young adults: A global analysis based on the GBD 2021 and a value of statistical life year framework

**DOI:** 10.1111/dme.70255

**Published:** 2026-02-13

**Authors:** Hang Guo, Zhaoyu Guo, Yunfei Liu, Quan Zhang

**Affiliations:** ^1^ NHC Key Lab of Hormones and Development and Tianjin Key Lab of Metabolic Diseases Tianjin Medical University Chu Hsien‐I Memorial Hospital & Institute of Endocrinology Tianjin China; ^2^ Oxford Centre for Diabetes, Endocrinology and Metabolism University of Oxford Oxford UK; ^3^ Big Data Institute, Nuffield Department of Population Health University of Oxford Oxford UK; ^4^ Department of Mechanical Engineering Tsinghua University Beijing China; ^5^ Mathematical Institute University of Oxford Oxford UK; ^6^ Center for Neuroscience and Cell Biology (CNC), Centre for Innovative Biomedicine and Biotechnology (CIBB) University of Coimbra Coimbra Portugal

**Keywords:** diabetes, disability‐adjusted life years, global health, health economics

## Abstract

**Aims:**

The rising prevalence of diabetes in young adults threatens global health and sustainable development. However, its full macroeconomic impact, especially the welfare losses beyond conventional productivity costs, has not been systematically quantified at a global level. We aimed to estimate the current and future global economic burden of diabetes in individuals aged 15–39 years using a welfare‐based approach.

**Methods:**

Using disability‐adjusted life‐years (DALYs) from the Global Burden of Disease Study 2021, we monetized health losses into a value of lost welfare (VLW) via a value of a statistical life year approach. We estimated the VLW globally, regionally and nationally, analysed its distribution by Socio‐Demographic Index (SDI) and made projections to 2050.

**Results:**

In 2021, the global economic burden was Int$1.16 trillion. Low‐SDI regions had the highest relative burden (1.21% of GDP), whereas high‐SDI regions had the largest absolute loss. Geographically, East Asia showed the highest absolute burden (Int$240.30 billion) and Oceania the highest relative impact (3.03% of GDP). Projections show the burden will more than double by 2050 to 1.32% of global GDP, driven almost entirely by type 2 diabetes.

**Conclusions:**

Diabetes in young adults represents a significant and growing macroeconomic burden. Our findings underscore the urgent need for public health strategies and economic policies focused on preventing type 2 diabetes. Such interventions are especially critical for countries with fragile health systems to mitigate substantial future economic and health losses.

**Key Points**

- Using Global Burden of Disease data, we quantify a substantial global economic burden of diabetes among young adults (15–39 years), amounting to 0.76% of global GDP in 2021. Without effective intervention, this burden is projected to rise to 0.87% by 2050.
- Type 2 diabetes is the dominant driver of this economic loss in young populations.
- Profound inequities exist: although high‐income countries sustain the largestabsolute monetary losses, the relative economic impact is disproportionatelysevere in low‐income regions and Small Island Developing States.
- These findings provide urgent economic justification for prioritising earlyprevention and intervention to curb the rising burden of young‐onset diabetes.

## 1 INTRODUCTION

Diabetes has become one of the most formidable global public health challenges of the 21st century.^1^ As a complex chronic metabolic disorder, it is characterized by persistent hyperglycaemia, which can lead to severe complications, posing a long‐term threat to individual health and quality of life.^2^ Globally, diabetes is not merely a clinical issue but a public health imperative that profoundly impacts socio‐economic development.^3^ Over the past three decades, driven by population growth, ageing and urbanization, the prevalence of type 2 diabetes has grown substantially.^4^ Alarmingly, this epidemic is increasingly affecting younger populations, shattering the traditional perception of it as a disease of middle and older age.^5^

Despite advances in care, the burden of diabetes remains poorly controlled.^6^ The impact on young adults (15–39 years) is particularly underestimated. This group is the backbone of the workforce, representing the peak phase of human capital accumulation—spanning higher education, career establishment and family formation. Disease onset during this window disrupts these critical developmental trajectories, resulting in compounded economic consequences that extend decades into the future.^7^ However, most current research and public health policies focus on older patients, leaving a significant knowledge gap regarding the economic burden in young adults.^8^ Thus, the true societal cost of diabetes is often underestimated and intervention strategies incomplete.

The Global Burden of Disease (GBD) study's primary metric, the disability‐adjusted life‐year (DALY), is invaluable for measuring health loss but has limitations. As a non‐monetary metric, it struggles to convey the full economic impact to policymakers, especially in finance ministries, making it difficult to justify health investments against competing priorities like education or infrastructure.^9^, ^10^ In contrast, the value of lost welfare (VLW) model directly monetizes health losses, providing a clear link between health outcomes and fiscal policy considerations.^11^ By integrating DALYs with the value of a statistical life (VSL), it quantifies the monetary value of disability and death, including intangible costs like pain and suffering.^12^ This comprehensive approach is advocated by the World Health Organization (WHO) and has been used for other diseases.^13^ However, such a systematic economic assessment has not been conducted for diabetes, particularly in young adults.

Therefore, this study uses GBD 2021 data and the value of a statistical life year (VSLY) method to estimate the economic burden of diabetes in young adults (15–39 years) at global, regional and national levels for 2021, with projections to 2050. By providing a monetized metric, we aim to reveal the true macroeconomic impact of diabetes and offer robust evidence for governments to formulate cost‐effective prevention strategies, optimize resource allocation and address this growing epidemic.

## 2 METHODS

### 2.1 Data sources

This study draws on multiple standardized global databases. The data were obtained from the GBD 2021 published by the Institute for Health Metrics and Evaluation (available at https://ghdx.healthdata.org/). GBD 2021 reports DALYs, life expectancy and population size stratified by age, sex and region. Specifically, diabetes‐related DALYs in the GBD framework aggregate Years of Life Lost and Years Lived with Disability, derived from cause‐specific mortality and prevalence data, respectively.

Data on GDP and per capita GDP, measured in purchasing power parity at constant 2021 international dollars, were obtained from the World Bank's World Development Indicators database. The baseline value for the 2021 US VSL was sourced from official estimates by the US Department of Transportation.^14^, ^15^ All results are presented in 2021 international dollars, adjusted for purchasing power parity.^16^

### 2.2 VLW and VLW/GDP

This study adopted the VSLY approach to monetize health losses, which were initially measured in DALYs. The method operates on the principle that any loss of a healthy life year has a corresponding, measurable economic or social cost. This monetization strategy is well established in the global health economics literature as a means to rationalize policy prioritization and resource allocation decisions.^11^, ^13^, ^17^, ^18^

The VSL for each country (VSL country) was estimated using a benefit transfer model, with the US VSL as the reference point. The choice of the reference benchmark is based on the consideration that the United States possesses the most extensive and robust research data on willingness‐to‐pay to determine the VSL. By employing the benefit transfer method and using income elasticity to adjust the baseline value according to each country's per capita GDP, we were able to estimate VSLs that are commensurate with each nation's economic level. This ensures that the valuation is both globally comparable and reflective of national economic realities. The calculation followed the formula:

$$\mathrm{VSL}i=\text{VSLUSA}\times {\left(\frac{\mathrm{GDP}i}{\text{GDPUSA}}\right)}^{\mathrm{IE}}$$

where ${\mathrm{VSL}}_{\mathrm{USA}}=\mathrm{US}11.8$ million, based on 2021 valuations (https://www.transportation.gov/office‐policy/transportation‐policy/revised‐departmental‐guidance‐on‐valuation‐of‐a‐statistical‐life‐in‐economic‐analysis), and IE denotes the income elasticity of the VSL. A base‐case elasticity of 1.0 was assumed, implying a proportional relationship between a country's per capita GDP and its VSL. Country‐level GDP was defined as gross domestic product per capita at purchasing power parity (PPP), in constant 2021 international dollars.

The model rests on several key assumptions:

- VSL is determined solely by economic development, disregarding potential influences of culture, legal environment, or health system structure.
- Country‐level GDP values are static during the estimation period.
- The benefit transfer model assumes cross‐national comparability of income elasticity in valuing life.

To calculate the VSLY, the country‐specific VSL was annualized using the approximation of future life expectancy (FLE):

$$\text{VSLYi}=\frac{\text{VSLi}}{{\mathrm{FLE}}_{i}}.$$

The estimation of FLE is based on the following assumptions.

### 2.3 FLE approximation

FLE is approximated as half of the Health‐Adjusted Life Expectancy (FLE ≈ HALE/2). Thereby assuming that each DALY represents the loss of half a healthy life year. A single‐year HALE estimate was applied uniformly across the study period for all countries.

### 2.4 Sensitivity analyses

To assess robustness, sensitivity analyses with IE values of 0.55, 1.0 and 1.5 were also conducted to assess how variations in willingness to pay, driven by different income levels, influence the results. This approach ensures that the estimates remain relevant for policy considerations across diverse economic contexts.^11^, ^13^, ^19^

### 2.5 Estimating total economic loss (VLW)

Total economic loss, expressed as the VLW, was calculated by multiplying DALYs with the corresponding VSLY:

$$\mathrm{VLW}i=\text{DALYs}i\times {\text{VSLY}}_{i}$$

DALY data were extracted as median estimates along with 95% uncertainty intervals from the GBD 2021 dataset. For the purposes of this analysis, DALY estimates were assumed to be unbiased and comparable across countries and regions, based on standardized GBD estimation methods.

### 2.6 Expressing VLW as a share of GDP

To evaluate the relative economic impact of health loss across settings, VLW was expressed as a percentage of country‐level GDP:

$$\mathrm{GDP}i*\left(\%\right)=\left(\frac{\mathrm{VLW}i}{{\mathrm{GDP}}_{i}}\right)\times 100\backslash \%.$$

This metric enables direct comparison of macroeconomic burden across heterogeneous economies, adjusting for differences in national output. All GDP values refer to total PPP‐adjusted GDP (in constant 2021 international dollars) for each country. The VLW and GDP (VLW) for GBD 2021 regions are also considered.

### 2.7 Statistical analysis

All data processing and computational analyses were executed within the RStudio Integrated Development Environment (RStudio IDE, RStudio PBC, Boston, MA, USA). In instances of missing 2021 macroeconomic data for certain locales, values were imputed using a linear regression model. Furthermore, the 95% uncertainty intervals (UIs) reported with the GBD 2021 DALY estimates were incorporated into our valuation model (shown as error bars in figures) to produce corresponding uncertainty intervals for the VSLY estimates. The research framework was structured in reference to best practice principles for reporting health economic evaluations.

## 3 RESULTS

### 3.1 Global trend

Globally, the total economic burden of diabetes in young adults aged 15–39 years was estimated at Int$1,159,179.45 million, which accounted for 0.76% of global GDP. Type 2 diabetes was the principal contributor to this economic loss, with a total VLW of Int$929,240.25 million, equivalent to 0.61% of global GDP. The economic burden attributable to type 1 diabetes was estimated at Int$229,939.21 million, representing 0.15% of global GDP (Table 1).

**TABLE 1:** VLW and VLW/GDP by GBD regions in 2021 for diabetes in young adults, generated using income elasticity of the VSL at 1.00.

	Overall diabetes		Type 2 diabetes		Type 1 diabetes	
	VLW region (millions)	VLW/GDP (%)	VLW region (millions)	VLW/GDP (%)	VLW region (millions)	VLW/GDP (%)
Globa	1159179.45	0.76	929240.25	0.61	229939.21	0.15
High SDI	373302.01	0.62	272196.39	0.45	101105.61	0.17
High‐middle SDI	353815.96	0.70	307198.12	0.61	46617.84	0.09
Low SDI	22937.20	1.21	18309.78	0.97	4627.42	0.24
Low‐middle SDI	210738.12	1.07	171497.58	0.87	39240.54	0.20
Middle SDI	198386.17	0.96	160038.37	0.77	38347.80	0.18
Andean Latin America	5137.95	0.56	4446.51	0.49	691.45	0.08
Australasia	4728.96	0.27	2497.72	0.14	2231.24	0.13
Caribbean	9396.46	1.56	7596.78	1.26	1799.67	0.30
Central Asia	12757.04	0.86	9200.85	0.62	3556.19	0.24
Central Europe	14616.40	0.35	9522.84	0.23	5093.56	0.12
Central Latin America	66126.13	1.53	55781.80	1.29	10344.32	0.24
Central Sub‐Saharan Africa	6817.22	1.48	5706.59	1.24	1110.62	0.24
East Asia	240296.20	0.83	225492.05	0.78	14804.15	0.05
Eastern Europe	37900.14	0.54	22848.80	0.33	15051.35	0.22
Eastern Sub‐Saharan Africa	11683.03	1.00	8499.99	0.73	3183.04	0.27
High‐income Asia Pacific	45551.77	0.51	41008.22	0.46	4543.55	0.05
High‐income North America	174298.15	0.67	108778.02	0.42	65520.13	0.25
North Africa and Middle East	126349.20	1.12	109090.39	0.96	17258.81	0.15
Oceania	1975.79	3.03	1791.83	2.74	183.95	0.28
South Asia	152968.02	1.09	123768.76	0.88	29199.26	0.22
Southeast Asia	67833.45	0.76	54295.20	0.61	13538.25	0.15
Southern Latin America	7387.72	0.40	5163.44	0.28	2224.28	0.12
Southern Sub‐Saharan Africa	11796.20	1.29	9997.74	1.10	1798.45	0.20
Tropical Latin America	31914.67	0.78	22704.79	0.56	9209.88	0.26
Western Europe	108961.58	0.45	83496.44	0.34	25465.14	0.11
Western Sub‐Saharan Africa	20683.38	0.92	17551.47	0.78	3131.91	0.14

Abbreviations: GBD, Global Burden of Disease; GDP, gross domestic product; SDI, Socio‐Demographic Index; T1DM, type 1 diabetes mellitus; T2DM, type 2 diabetes mellitus; VLW, value of lost welfare.

### 3.2 SDI regions trends

**FIGURE 1:**
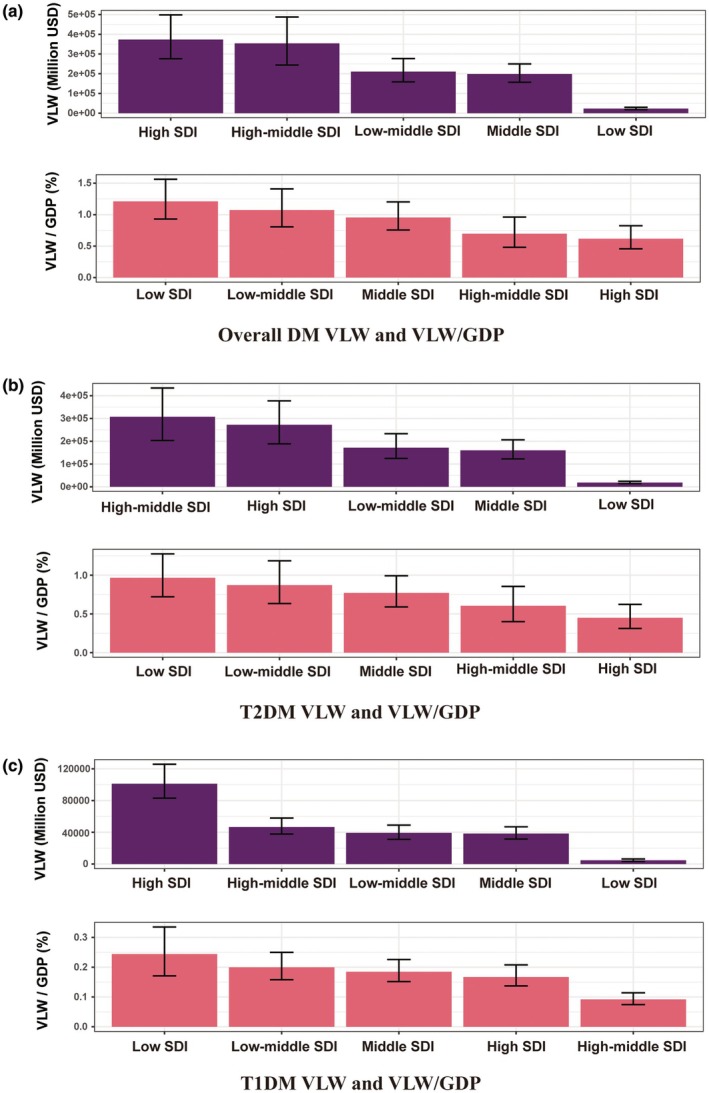
VLW and VLW/GDP by five SDI regions in 2021 for diabetes in young adults, generated using income elasticity of the VSL at 1.00. (a) Overall DM VLW and VLW/GDP, (b) T2DM VLW and VLW/GDP, (c) T1DM VLW and VLW/GDP. Error bars were derived primarily from the 95% uncertainty intervals of DALY estimates. DM, diabetes mellitus; GDP, gross domestic product; SDI, Socio‐Demographic Index; T1DM, type 1 diabetes mellitus; T2DM, type 2 diabetes mellitus; VLW, value of lost welfare; VSL, value of statistical life.

Diabetes‐related economic losses exhibit significant disparities across different Socio‐Demographic Index (SDI) regions. The VLW of overall diabetes in young adults aged 15–39 years was greatest in high‐SDI (Int$373,302.01 million). Conversely, the relative economic burden, as a percentage of GDP, was highest in low‐SDI regions (1.21%) and showed a clear inverse relationship with SDI, being lowest in high‐SDI regions (0.62%) (Table 1, Figure 1a).

The absolute VLW for type 2 diabetes in young adults aged 15–39 years was highest in high‐middle‐SDI regions (Int$307,198.12 million), whereas the relative burden was greatest in low‐SDI regions (0.97%) and lowest in high‐SDI regions (0.45%) (Table 1, Figure 1b).

For type 1 diabetes in young adults aged 15–39 years, the highest absolute economic burden was concentrated in high‐SDI regions (Int$101,105.61 million). The relative burden as a percentage of GDP was again highest in low‐SDI regions (0.24%) (Table 1, Figure 1c).

### 3.3 Geographical regions trends

**FIGURE 2:**
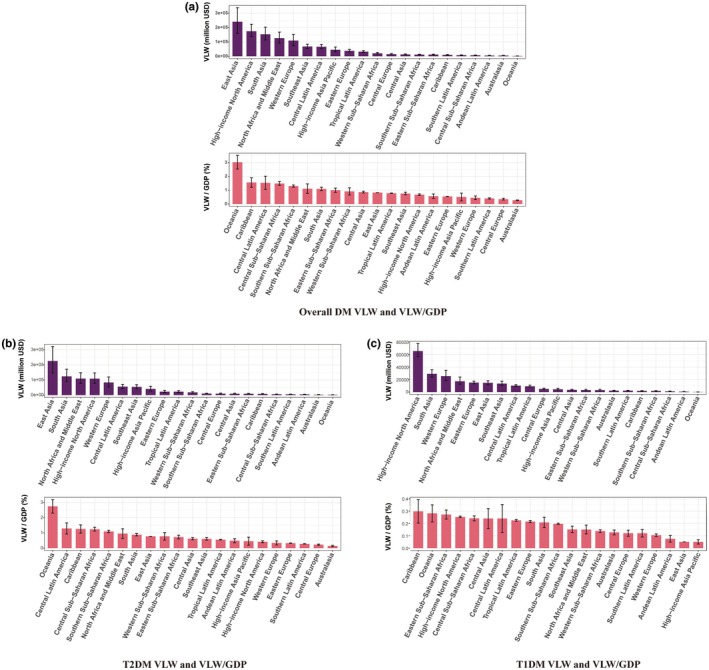
VLW and VLW/GDP by GBD geographic regions in 2021 for diabetes in young adults, generated using income elasticity of the VSL at 1.00. (a) Overall DM VLW and VLW/GDP, (b) T2DM VLW and VLW/GDP, (c) T1DM VLW and VLW/GDP. Error bars were derived primarily from the 95% uncertainty intervals of DALY estimates. GBD, Global Burden of Disease; GDP, gross domestic product; SDI, Socio‐Demographic Index; T1DM, type 1 diabetes mellitus; T2DM, type 2 diabetes mellitus; VLW, value of lost welfare; VSL, value of statistical life.

Analysis across 21 geographical regions revealed substantial variation in the economic burden of diabetes. For overall diabetes, the absolute economic burden, measured as the VLW, was highest in East Asia (Int$240,296.20 million). The lowest absolute burden was observed in Oceania (Int$1975.79 million). When expressed as a proportion of GDP, the pattern was different, with the highest relative burden found in Oceania (3.025%). Conversely, the lowest relative burdens were in Australasia (0.270%) (Table 1, Figure 2a).

Type 2 diabetes drove the majority of the overall burden. The VLW was highest in East Asia (Int$225,492.05 million). The lowest absolute burden was observed in Oceania (Int$1791.83 million). When expressed as a proportion of GDP, the pattern was different, with the highest relative burden found in Oceania (2.743%). Conversely, the lowest relative burdens were in Australasia (0.143%) (Table 1, Figure 2b).

For type 1 diabetes, the absolute economic burden measured as the VLW was highest in High‐income North America (Int$65,520.13 million). The lowest absolute burden was observed in Oceania (Int$183.95 million). When expressed as a proportion of gross domestic product (GDP), the pattern was different, with the highest relative burden found in the Caribbean (0.298%) (Table 1, Figure 2c).

### 3.4 National Trends

**FIGURE 3:**
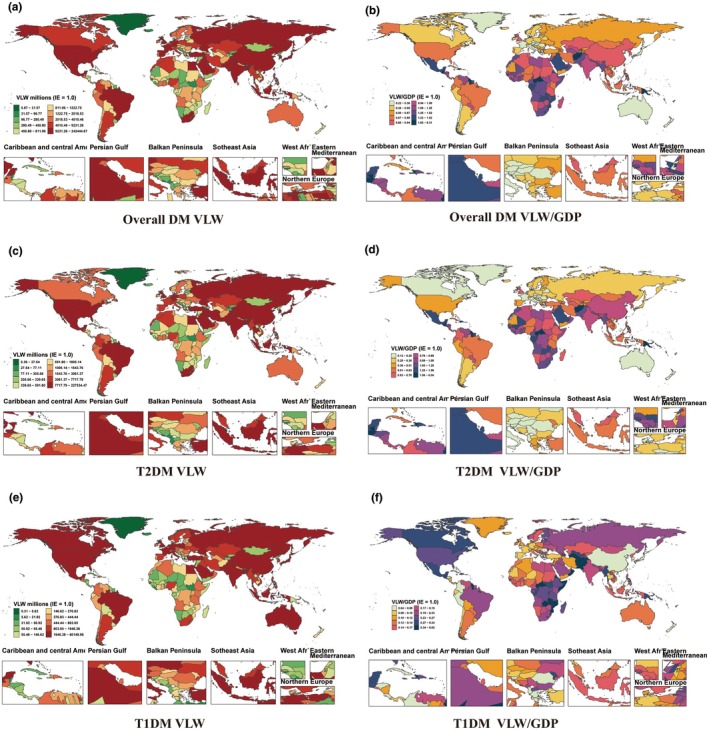
VLW and VLW/GDP by GBD countries and territories in 2021 for diabetes in Young Adults, generated using income elasticity of the VSL at 1.00. (a) Overall DM VLM, (b) overall DM VLW/GDP, (c) T2DM VLW, (d) T2DM VLW/GDP, (e) T1DM VLW, (f) T1DM VLW/GDP. GBD, Global Burden of Disease; GDP, gross domestic product; DM, diabetes mellitus; VLW, value of lost welfare; VSL, value of statistical life.

At the national level, the absolute economic burden (VLW) of overall diabetes was highest in China (Int$240,044.23 million), the United States (Int$165,319.85 million) and India (Int$117,077.34 million). In stark contrast, the relative burden as a percentage of GDP was most severe in several Oceanian nations, including the Marshall Islands (6.24%), Kiribati (4.41%) and Fiji (4.13%). The lowest relative burdens were observed in several Central and Eastern European countries, including Romania, Albania and Slovenia (all 0.23%) (Figure 3a,b, Supplemental Table S1).

The burden of type 2 diabetes largely drove the overall patterns. China experienced the highest absolute VLW (Int$225,281.66 million). The relative impact was again most severe in the Marshall Islands. High‐income countries such as Australia reported some of the lowest relative burdens (0.13%) for type 2 diabetes (Figure 3c,d, Supplemental Table S1).

For type 1 diabetes, the United States shouldered the highest absolute economic loss by a substantial margin (Int$59,554.42 million). The relative burden, however, was most significant in Haiti (0.81%). Several high‐income Asian countries, including Japan and Singapore (both 0.04%), recorded the lowest relative burdens globally (Figure 3e,f, Supplemental Table S1).

### 3.5 Predictions of diabetes economic burden

**FIGURE 4:**
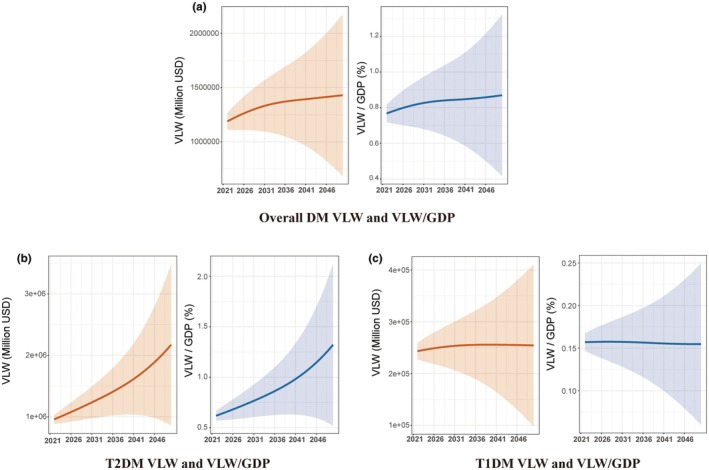
The projections of the economic burden for diabetes from 2021 to 2050, generated using income elasticity of the VSL at 1.00. (a) Overall DM VLW and VLW/GDP, (b) T2DM VLW and VLW/GDP, (c) T1DM VLW and VLW/GDP. DM, diabetes mellitus; GDP, gross domestic product; T1DM, type 1 diabetes mellitus; T2DM, type 2 diabetes mellitus; VLW, value of lost welfare; VSL, value of statistical life.

Projections to 2050 indicate a substantial and growing economic burden from diabetes in young adults, driven almost entirely by type 2 diabetes. The global economic burden (VLW) of overall diabetes is projected to increase from Int$1,188,874.80 million in 2022 to Int$1,429,575.56 million by 2050. The relative burden is also forecast to rise steadily, from 0.77% of global GDP in 2022 to 0.87% by 2050 (Figure 4a, Supplemental Table S2).

The increase in the overall burden is overwhelmingly driven by type 2 diabetes. Its absolute VLW is forecast to more than double, escalating from Int$958,726.99 million in 2022 to Int$2,172,379.75 million in 2050. Consequently, its relative economic burden is also projected to more than double, rising from 0.62% of GDP in 2022 to 1.32% by 2050 (Figure 4b, Supplemental Table S2).

In stark contrast, the economic burden attributable to type 1 diabetes is projected to remain relatively stable. The absolute VLW is forecast to peak at Int$255,650.94 million in 2039 and remain near that level through 2050, while the relative burden is expected to stay constant at approximately 0.15%–0.16% of GDP throughout the projection period (Figure 4c, Supplemental Table S2).

### 3.6 Sensitivity analysis

Estimates of VLW and VLW/GDP for diabetes in 2021 by GBD region, countries and territories, using IEs of 0.55 and 1.50, are presented in the supplementary tables (Supplemental Tables S3–, S6).

## 4 DISCUSSION

This study represents the first comprehensive economic evaluation of the diabetes burden specifically targeting the critical yet historically overlooked youth population aged 15–39 years. By employing a welfare‐based valuation approach, we reveal that the global diabetes‐related economic burden in this demographic reached Int$1.16 trillion in 2021, accounting for 0.76% of global GDP, with type 2 diabetes as the dominant driver. Beyond the aggregate magnitude, our analysis uncovers a profound ‘economic paradox’: although high‐income countries incur the largest monetary losses, the actual economic strain is far more severe in low‐income regions and Small Island Developing States. By quantifying the erosion of human capital in this pivotal ‘demographic dividend’ phase, our findings suggest early‐onset diabetes is not only a clinical challenge but also a systemic threat to sustainable development, providing compelling economic justification for prioritizing early intervention.

This is the first study to employ the VSLY method—grounded in welfare economics theory, in conjunction with standardized data from the Global Burden of Diseases, Injuries and Risk Factors Study 2021. Traditional models, such as the Cost‐of‐Illness and Human Capital Approach, primarily measure economic losses in terms of lost output or direct medical expenditures.^20^ A fundamental limitation of these approaches is the equation of human value with market productivity, a perspective that neglects the health value of individuals outside the labour force and ignores non‐market losses (such as pain, suffering and diminished quality of life) associated with disease, thereby raising ethical concerns. In contrast, the VSLY framework adopted in this study overcomes these limitations by capturing the comprehensive value of healthy life years lost, providing a robust yet conservative estimate of the societal welfare burden.^21^ Operating on the ‘willingness‐to‐pay’ principle, the VSLY model quantifies the monetary amount society is willing to pay to avert the loss of a single healthy life year.^22^ This person‐centred approach offers a more comprehensive assessment by monetizing the total welfare loss resulting from premature mortality and disability.^23^, ^24^ Furthermore, it provides a consistent metric for decision‐making, facilitating cross‐sectoral cost–benefit analyses. Comparing our results with studies utilizing identical methodology reveals the disproportionate economic impact of early‐onset diabetes. Although the total global burdens of stroke (1.66% of GDP)^11^ and musculoskeletal disorders (1.41%)^13^ encompass all ages, diabetes in young adults (15–39) alone accounts for 0.76% of global GDP. Remarkably, welfare losses in this single demographic are equivalent to approximately half the total global burden of these major conditions, highlighting the severe impact of early‐onset diabetes on human capital. Collectively, these findings demonstrate that the VSLY model reveals the true societal cost of disease with greater depth and scope, providing powerful economic evidence to inform policymaking.^25^

Regarding disease drivers, type 2 diabetes serves as the primary force behind the economic burden in young adults, substantially outweighing type 1. This disparity is rooted in their distinct etiological profiles: whereas type 1 diabetes is an autoimmune disorder with relatively stable incidence,^26^ the type 2 epidemic is driven by modifiable risk factors that have been rapidly exacerbated by global lifestyle shifts. Notably, however, our analysis indicates that type 1 diabetes accounts for nearly 20% of the total economic burden in this specific cohort (15–39 years), a proportion significantly higher than the 5%–10% typically observed in all‐age studies. This difference arises because the mortality and disability burden of early‐onset type 1 diabetes is concentrated in childhood and young adulthood, whereas the peak burden of type 2 diabetes remains in middle and older age groups that are not included in this analysis.^1^ Nevertheless, given that type 2 diabetes is the dominant driver of future growth in economic burden and is largely preventable, prioritizing targeted primary prevention strategies for this subtype represents the most cost‐effective approach to mitigating substantial future economic losses.

The economic burden of diabetes presents a stark paradox across different levels of socio‐economic development. Although high‐SDI countries shoulder the greatest absolute economic loss—reflecting their higher per capita GDP—the relative burden is most severe in low‐SDI regions. Our data indicate that diabetes consumes 1.21% of GDP in low‐SDI countries, nearly double the proportion in high‐SDI nations (0.62%). This inequality extends beyond immediate fiscal strain; it threatens to entrench global economic disparities. For developing nations, the erosion of human capital in this critical age group (15–39 years) threatens to undermine the potential ‘demographic dividend’, creating a vicious cycle where poor health constrains economic growth and, in turn, limits investment in health.^27^ Furthermore, in resource‐constrained settings, such substantial economic burdens may crowd out investments in other essential sectors, such as education and infrastructure, thereby widening the developmental chasm between the Global North and South.

Our study reveals a profound dichotomy between absolute and relative economic burdens across geographical and national scales. Whereas the highest absolute losses are concentrated in major economies in East Asia, South Asia and North America—driven by China, India and the United States—the most severe relative burden falls upon smaller economies in Oceania and the Caribbean. Specifically, Small Island Developing States like the Marshall Islands (6.24%) and Kiribati (4.41%) experience disease costs that consume an exceptionally high share of GDP, posing substantial challenges to economic sustainability. This divergence persists across subtypes: type 2 diabetes is concentrated in Asian regions, whereas type 1 imposes high absolute costs in wealthy nations but critical relative challenges in resource‐limited settings. These findings underscore the need for global health organizations to recognize such vulnerability and provide targeted technical and financial support to enable context‐specific, precision interventions across diverse economic settings.^3^, ^28^

Our projections to 2050 indicate that the economic burden of diabetes in young adults will continue to increase globally, driven almost entirely by type 2 diabetes. By 2050, the relative burden of type 2 diabetes is projected to double from 0.62% in 2022 to 1.32%. The drivers of this trend are persistent urbanization, the growing prevalence of obesity and sedentary lifestyles worldwide. In contrast, the burden of type 1 diabetes is projected to remain stable, which is consistent with its nature as an autoimmune disease whose incidence is relatively unaffected by macroeconomic lifestyle trends.

This study has several limitations. First, our estimates' validity depends on GBD 2021 data, which has uncertainty for data‐sparse regions. Second, our VSL estimation uses a benefit transfer approach that may not fully capture national heterogeneity. Finally, our model employed static 2021 values for GDP and HALE, failing to account for their dynamic evolution over time.

In conclusion, this study, using the innovative VSLY method, reveals a profoundly unequal distribution of the economic burden of diabetes among young adults aged 15–39 years. It finds that the vast majority of this burden is driven by type 2 diabetes, which disproportionately undermines the economic development potential of low‐SDI countries and specific geographic regions. Furthermore, the economic burden of diabetes in young adults globally is projected to continue its growth towards 2050. Our findings provide a powerful economic rationale for global policymakers. The results underscore the urgency of integrating diabetes control into global health and economic policies, calling for increased financial support in high‐burden regions.

## AUTHOR CONTRIBUTIONS

QZ and HG conceived and designed the manuscript; ZY‐G did a literature search and downloaded the data. QZ, HG, ZY‐G and YF‐L conducted the analysis and interpretation of the data. HG drafted the manuscript. HG, ZY‐G and YF‐L compiled the tables and figures.

QZ are the corresponding authors. All authors participated in data analysis, interpretation, discussion and writing of the manuscript, and all authors read and approved the final version of the paper.

## FUNDING INFORMATION

This work was supported by the National Natural Science Foundation of China (no. 82305007), China Postdoctoral Science Foundation (no. 2024M762380), National Funds via FCT under project UIDP/04539/2020 (QZ), the FEDER within the scope of COMPETE 2030 and by National Funds via FCT (MPr‐2023‐12‐SACCCT‐Projetos de IC&DT Programme, COMPETE2030‐FEDER‐00698600, https://doi.org/10.54499/2023.16678.ICDT, QZ).

## CONFLICT OF INTEREST STATEMENT

No potential conflict of interest was reported by the author(s).

## ETHICS STATEMENT

The protocol of the GBD 2021 has been approved by the research ethics board at the University of Washington. The GBD 2021 shall be conducted in full compliance with University of Washington policies and procedures as well as applicable federal, state and local laws.

## Supporting information


**Table S1.** VLW and VLW/GDP by GBD countries and territories in 2021 for diabetes in young adults, generated using income elasticity of the VSL at 1.00.

**Table S2.** The projections of the economic burden for diabetes from 2021 to 2050, generated using income elasticity of the VSL at 1.00.

**Table S3.** VLW and VLW/GDP by GBD regions in 2021 for diabetes in young adults, generated using income elasticity of the VSL at 0.55.

**Table S4.** VLW and VLW/GDP by GBD regions in 2021 for diabetes in young adults, generated using income elasticity of the VSL at 1.5.

**Table S5.** VLW and VLW/GDP by GBD countries and territories in 2021 for diabetes in young adults, generated using income elasticity of the VSL at 0.55.

**Table S6.** VLW and VLW/GDP by GBD countries and territories in 2021 for diabetes in young adults, generated using income elasticity of the VSL at 1.5.

## Data Availability

The datasets analysed during the current study are available at https://vizhub.healthdata.org/gbd‐results and the World Bank's World Development Indicators database (https://databank.worldbank.org/databases).

## References

[1] Global, regional, and national burden of diabetes from 1990 to 2021, with projections of prevalence to 2050: a systematic analysis for the global burden of disease study 2021. Lancet. 2023;402:203‐234. 37356446 10.1016/S0140-6736(23)01301-6 PMC10364581

[2] Abel ED , Gloyn AL , Evans‐Molina C , et al. Diabetes mellitus‐Progress and opportunities in the evolving epidemic. Cell. 2024;187:3789‐3820. 39059357 10.1016/j.cell.2024.06.029 PMC11299851

[3] Bommer C , Heesemann E , Sagalova V , et al. The global economic burden of diabetes in adults aged 20‐79 years: a cost‐of‐illness study. Lancet Diabetes Endocrinol. 2017;5:423‐430. 28456416 10.1016/S2213-8587(17)30097-9

[4] Soares Andrade CA , Shahin B , Dede O , et al. The burden of type 2 diabetes mellitus in states of the European Union and United Kingdom at the national and subnational levels: a systematic review. Obes Rev. 2023;24:e13593. 37401729 10.1111/obr.13593

[5] Luk A , Wild SH , Jones S , et al. Early‐onset type 2 diabetes: the next major diabetes transition. Lancet. 2025;405:2313‐2326. 40570862 10.1016/S0140-6736(25)00830-X

[6] Chen X , Zhang L , Chen W . Global, regional, and national burdens of type 1 and type 2 diabetes mellitus in adolescents from 1990 to 2021, with forecasts to 2030: a systematic analysis of the global burden of disease study 2021. BMC Med. 2025;23:48. 39876009 10.1186/s12916-025-03890-w PMC11776159

[7] Perng W , Conway R , Mayer‐Davis E , Dabelea D . Youth‐onset type 2 diabetes: the epidemiology of an awakening epidemic. Diabetes Care. 2023;46:490‐499. 36812420 10.2337/dci22-0046 PMC10090267

[8] Parker ED , Lin J , Mahoney T , et al. Economic costs of diabetes in the U.S. in 2022. Diabetes Care. 2024;47:26‐43. 37909353 10.2337/dci23-0085

[9] Global, regional, and national burden of disorders affecting the nervous system, 1990‐2021: a systematic analysis for the global burden of disease study 2021. Lancet Neurol. 2024;23:344‐381. 38493795 10.1016/S1474-4422(24)00038-3 PMC10949203

[10] Global, regional, and national prevalence of child and adolescent overweight and obesity, 1990‐2021, with forecasts to 2050: a forecasting study for the global burden of disease study 2021. Lancet. 2025;405:785‐812. 40049185 10.1016/S0140-6736(25)00397-6 PMC11920006

[11] Gerstl JVE , Blitz SE , Qu QR , et al. Global, regional, and National Economic Consequences of stroke. Stroke. 2023;54:2380‐2389. 37497672 10.1161/STROKEAHA.123.043131 PMC7614992

[12] Alkire BC , Shrime MG , Dare AJ , Vincent JR , Meara JG . Global economic consequences of selected surgical diseases: a modelling study. Lancet Glob Health. 2015;3(Suppl 2):S21‐S27. 25926317 10.1016/S2214-109X(15)70088-4 PMC4884437

[13] Qiu K , Wang C , Mo X , et al. The global macroeconomic burden of musculoskeletal disorders. Int J Surg. 2025;111:7857‐7866. 40694023 10.1097/JS9.0000000000003072 PMC12626537

[14] Chen XF , Li Q , Bergquist R , et al. Estimation and prediction on the economic burden of schistosomiasis in 25 endemic countries. Infect Dis Poverty. 2025;14:49. 40524274 10.1186/s40249-025-01330-8 PMC12168328

[15] Vidavalur R , More K , Bhutani VK . Assessment of global burden due to neonatal encephalopathy: an economic evaluation. Semin Fetal Neonatal Med. 2024;29:101560. 39537453 10.1016/j.siny.2024.101560

[16] Vardavas C , Zisis K , Nikitara K , et al. Cost of the COVID‐19 pandemic versus the cost‐effectiveness of mitigation strategies in EU/UK/OECD: a systematic review. BMJ Open. 2023;13:e077602. 10.1136/bmjopen-2023-077602 PMC10619092 37907290

[17] Global burden and strength of evidence for 88 risk factors in 204 countries and 811 subnational locations, 1990‐2021: a systematic analysis for the global burden of disease study 2021. Lancet. 2024;403:2162‐2203. 38762324 10.1016/S0140-6736(24)00933-4 PMC11120204

[18] Chen S , Cao Z , Prettner K , et al. Estimates and projections of the global economic cost of 29 cancers in 204 countries and territories from 2020 to 2050. JAMA Oncol. 2023;9:465‐472. 36821107 10.1001/jamaoncol.2022.7826 PMC9951101

[19] Ranganathan K , Singh P , Raghavendran K , et al. The global macroeconomic burden of breast cancer: implications for oncologic surgery. Ann Surg. 2021;274:1067‐1072. 32097168 10.1097/SLA.0000000000003662

[20] Jo C . Cost‐of‐illness studies: concepts, scopes, and methods. Clin Mol Hepatol. 2014;20:327‐337. 25548737 10.3350/cmh.2014.20.4.327 PMC4278062

[21] Kazi DS , Elkind MSV , Deutsch A , et al. Forecasting the economic burden of cardiovascular disease and stroke in the United States through 2050: a presidential advisory from the American Heart Association. Circulation. 2024;150:e89‐e101. 38832515 10.1161/CIR.0000000000001258

[22] Fan CY , Fann JC , Yang MC , et al. Estimating global burden of COVID‐19 with disability‐adjusted life years and value of statistical life metrics. J Formos Med Assoc. 2021;120(Suppl 1):S106‐s117. 34119392 10.1016/j.jfma.2021.05.019 PMC8165085

[23] Corlew DS , Alkire BC , Poenaru D , Meara JG , Shrime MG . Economic valuation of the impact of a large surgical charity using the value of lost welfare approach. BMJ Glob Health. 2016;1:e000059. 10.1136/bmjgh-2016-000059 PMC5321371 28588975

[24] Williams R , Karuranga S , Malanda B , et al. Global and regional estimates and projections of diabetes‐related health expenditure: results from the international diabetes federation diabetes atlas, 9th edition. Diabetes Res Clin Pract. 2020;162:108072. 32061820 10.1016/j.diabres.2020.108072

[25] Parada‐Contzen MV . The value of a statistical life for risk‐averse and risk‐seeking individuals. Risk Anal. 2019;39:2369‐2390. 31108566 10.1111/risa.13329

[26] Subramanian S , Khan F , Hirsch IB . New advances in type 1 diabetes. BMJ. 2024;384:e075681. 38278529 10.1136/bmj-2023-075681

[27] Mao W , Yip CW , Chen W . Complications of diabetes in China: health system and economic implications. BMC Public Health. 2019;19:269. 30841928 10.1186/s12889-019-6569-8 PMC6414024

[28] Lascar N , Brown J , Pattison H , Barnett AH , Bailey CJ , Bellary S . Type 2 diabetes in adolescents and young adults. Lancet Diabetes Endocrinol. 2018;6:69‐80. 28847479 10.1016/S2213-8587(17)30186-9

