# Supplementary material for: Estimating the economic burden of diabetes in young adults: A global analysis based on the GBD 2021 and a value of statistical life year framework
Source: Diabet Med. 2026 Feb 13;43(4):e70255. doi: 10.1111/dme.70255 (PMC12982657; doi:10.1111/dme.70255)
Supplement: Supplementary file 1 — Table S1. VLW and VLW/GDP by GBD countries and territories in 2021 for diabetes in young adults, generated using income elasticity of the VSL at 1.00. [file DME-43-e70255-s005.docx]

**Supplemental Table 1** VLW and VLW/GDP by GBD countries and territories in 2021 for diabetes in Young Adults, generated using income elasticity of the VSL at 1.00.

|  | Overall Diabetes | | Type 2 diabetes | | Type 1 diabetes | |
| --- | --- | --- | --- | --- | --- | --- |
|  | VLW region (millions) | VLW/GDP(%) | VLW region (millions) | VLW/GDP(%) | VLW region (millions) | VLW/GDP(%) |
| United States of America | 165319.85 | 0.70 | 105765.43 | 0.45 | 59554.42 | 0.25 |
| China | 240044.23 | 0.83 | 225281.66 | 0.78 | 14762.57 | 0.05 |
| Democratic People's Republic of Korea | 251.97 | 0.87 | 210.39 | 0.72 | 41.57 | 0.14 |
| Cambodia | 854.93 | 0.81 | 668.03 | 0.63 | 186.90 | 0.18 |
| Indonesia | 25505.12 | 0.72 | 19565.87 | 0.55 | 5939.25 | 0.17 |
| Lao People's Democratic Republic | 592.06 | 0.99 | 464.86 | 0.78 | 127.20 | 0.21 |
| Malaysia | 7830.32 | 0.83 | 6700.14 | 0.71 | 1130.18 | 0.12 |
| Maldives | 72.62 | 0.74 | 60.80 | 0.62 | 11.82 | 0.12 |
| Myanmar | 4039.57 | 1.38 | 3152.44 | 1.08 | 887.13 | 0.30 |
| Philippines | 9225.10 | 0.92 | 7317.54 | 0.73 | 1907.56 | 0.19 |
| Sri Lanka | 3187.33 | 1.00 | 2741.86 | 0.86 | 445.48 | 0.14 |
| Thailand | 9150.83 | 0.68 | 7757.11 | 0.57 | 1393.73 | 0.10 |
| Timor-Leste | 56.38 | 0.59 | 44.32 | 0.46 | 12.06 | 0.13 |
| Socialist Republic of Viet Nam | 6697.72 | 0.55 | 5339.07 | 0.44 | 1358.66 | 0.11 |
| Fiji | 408.36 | 4.13 | 374.05 | 3.79 | 34.31 | 0.35 |
| Kiribati | 16.28 | 4.41 | 15.45 | 4.18 | 0.83 | 0.23 |
| Marshall Islands | 23.56 | 6.24 | 22.56 | 5.98 | 1.00 | 0.26 |
| Federated States of Micronesia | 12.52 | 3.11 | 11.75 | 2.92 | 0.77 | 0.19 |
| Papua New Guinea | 1277.27 | 3.07 | 1143.89 | 2.75 | 133.38 | 0.32 |
| Samoa | 34.23 | 2.58 | 32.72 | 2.46 | 1.51 | 0.11 |
| Solomon Islands | 56.04 | 3.26 | 50.24 | 2.92 | 5.79 | 0.34 |
| Tonga | 14.67 | 1.96 | 13.83 | 1.85 | 0.84 | 0.11 |
| Vanuatu | 23.88 | 2.43 | 22.23 | 2.27 | 1.65 | 0.17 |
| Armenia | 305.22 | 0.64 | 205.13 | 0.43 | 100.09 | 0.21 |
| Azerbaijan | 1693.89 | 0.80 | 1089.73 | 0.52 | 604.16 | 0.29 |
| Georgia | 506.00 | 0.74 | 353.63 | 0.52 | 152.37 | 0.22 |
| Kazakhstan | 5379.92 | 0.86 | 4371.03 | 0.70 | 1008.89 | 0.16 |
| Kyrgyzstan | 227.54 | 0.58 | 164.50 | 0.42 | 63.04 | 0.16 |
| Mongolia | 356.56 | 0.72 | 266.59 | 0.54 | 89.98 | 0.18 |
| Tajikistan | 287.91 | 0.71 | 193.91 | 0.48 | 94.01 | 0.23 |
| Turkmenistan | 976.16 | 1.15 | 593.85 | 0.70 | 382.31 | 0.45 |
| Uzbekistan | 3023.84 | 0.96 | 1962.49 | 0.62 | 1061.35 | 0.34 |
| Albania | 96.97 | 0.23 | 63.65 | 0.15 | 33.31 | 0.08 |
| Bosnia and Herzegovina | 250.38 | 0.41 | 158.51 | 0.26 | 91.87 | 0.15 |
| Bulgaria | 1024.89 | 0.52 | 589.75 | 0.30 | 435.14 | 0.22 |
| Croatia | 435.77 | 0.28 | 273.95 | 0.18 | 161.82 | 0.1 |
| Czech Republic | 1553.55 | 0.31 | 995.95 | 0.20 | 557.6 | 0.11 |
| Hungary | 1144.24 | 0.31 | 776.85 | 0.21 | 367.39 | 0.10 |
| North Macedonia | 249.76 | 0.52 | 137.80 | 0.29 | 111.96 | 0.23 |
| Montenegro | 67.95 | 0.46 | 35.7 | 0.24 | 32.25 | 0.22 |
| Poland | 6441.42 | 0.41 | 4286.18 | 0.27 | 2155.24 | 0.14 |
| Romania | 1646.68 | 0.23 | 1151.23 | 0.16 | 495.45 | 0.07 |
| Serbia | 860.15 | 0.41 | 509.26 | 0.24 | 350.89 | 0.17 |
| Slovakia | 627.44 | 0.30 | 390.90 | 0.19 | 236.54 | 0.11 |
| Slovenia | 217.21 | 0.23 | 153.11 | 0.16 | 64.10 | 0.07 |
| Belarus | 1199.09 | 0.47 | 629.07 | 0.24 | 570.02 | 0.22 |
| Estonia | 317.38 | 0.55 | 167.57 | 0.29 | 149.81 | 0.26 |
| Latvia | 451.20 | 0.65 | 276.70 | 0.40 | 174.50 | 0.25 |
| Lithuania | 616.80 | 0.49 | 332.91 | 0.27 | 283.89 | 0.23 |
| Republic of Moldova | 449.18 | 0.80 | 293.81 | 0.52 | 155.37 | 0.28 |
| Russian Federation | 30691.56 | 0.54 | 18386.5 | 0.33 | 12305.06 | 0.22 |
| Ukraine | 4174.94 | 0.54 | 2762.24 | 0.36 | 1412.7 | 0.18 |
| Brunei Darussalam | 706.99 | 2.00 | 546.72 | 1.55 | 160.27 | 0.45 |
| Japan | 18874 | 0.33 | 16595.07 | 0.29 | 2278.92 | 0.04 |
| Republic of Korea | 20749.08 | 0.83 | 18974.54 | 0.76 | 1774.54 | 0.07 |
| Singapore | 5221.71 | 0.69 | 4891.89 | 0.65 | 329.82 | 0.04 |
| Australia | 3919.19 | 0.26 | 1882.21 | 0.13 | 2036.97 | 0.14 |
| New Zealand | 809.77 | 0.33 | 615.50 | 0.25 | 194.26 | 0.08 |
| Andorra | 22.60 | 0.44 | 16.57 | 0.33 | 6.03 | 0.12 |
| Austria | 1658.01 | 0.29 | 1088.18 | 0.19 | 569.83 | 0.10 |
| Belgium | 3170.89 | 0.46 | 2550.73 | 0.37 | 620.16 | 0.09 |
| Cyprus | 390.64 | 0.59 | 266.78 | 0.40 | 123.86 | 0.19 |
| Denmark | 1295.83 | 0.32 | 867.52 | 0.21 | 428.31 | 0.10 |
| Finland | 2064.66 | 0.66 | 1249.10 | 0.40 | 815.56 | 0.26 |
| France | 9824.39 | 0.28 | 6973.18 | 0.20 | 2851.21 | 0.08 |
| Germany | 20904.34 | 0.39 | 15407.68 | 0.29 | 5496.66 | 0.10 |
| Greece | 1683.26 | 0.49 | 1458.88 | 0.43 | 224.37 | 0.07 |
| Iceland | 96.65 | 0.45 | 77.93 | 0.36 | 18.72 | 0.09 |
| Ireland | 2132.46 | 0.36 | 1348.19 | 0.23 | 784.27 | 0.13 |
| Israel | 1594.72 | 0.36 | 1204.63 | 0.27 | 390.09 | 0.09 |
| Italy | 8770.95 | 0.29 | 5198.54 | 0.17 | 3572.41 | 0.12 |
| Luxembourg | 400.99 | 0.46 | 322.37 | 0.37 | 78.61 | 0.09 |
| Malta | 142.38 | 0.57 | 104.50 | 0.42 | 37.87 | 0.15 |
| Netherlands | 4123.89 | 0.35 | 2854.71 | 0.24 | 1269.17 | 0.11 |
| Norway | 2280.76 | 0.47 | 1434.38 | 0.30 | 846.38 | 0.18 |
| Portugal | 2225.26 | 0.54 | 1896.87 | 0.46 | 328.38 | 0.08 |
| Spain | 9532.86 | 0.47 | 7700.93 | 0.38 | 1831.92 | 0.09 |
| Sweden | 2816.72 | 0.43 | 1864.63 | 0.29 | 952.10 | 0.15 |
| Switzerland | 3966.80 | 0.55 | 3386.38 | 0.47 | 580.42 | 0.08 |
| United Kingdom of Great Britain and Northern Ireland | 29828.06 | 0.86 | 26196.46 | 0.76 | 3631.60 | 0.11 |
| Argentina | 5101.12 | 0.43 | 3449.88 | 0.29 | 1651.24 | 0.14 |
| Chile | 1907.54 | 0.35 | 1447.7 | 0.26 | 459.84 | 0.08 |
| Uruguay | 379.07 | 0.38 | 265.87 | 0.27 | 113.20 | 0.11 |
| Canada | 8965.19 | 0.42 | 3003.76 | 0.14 | 5961.43 | 0.28 |
| Antigua and Barbuda | 28.51 | 1.24 | 22.26 | 0.97 | 6.25 | 0.27 |
| Commonwealth of the Bahamas | 202.12 | 1.78 | 158.95 | 1.4 | 43.17 | 0.38 |
| Barbados | 57.52 | 1.23 | 44.36 | 0.95 | 13.16 | 0.28 |
| Belize | 76.35 | 1.52 | 60.84 | 1.21 | 15.51 | 0.31 |
| Cuba | 320.71 | 0.67 | 289.38 | 0.60 | 31.33 | 0.06 |
| Dominica | 18.52 | 1.77 | 15.42 | 1.47 | 3.10 | 0.30 |
| Dominican Republic | 3606.56 | 1.49 | 3032.71 | 1.26 | 573.85 | 0.24 |
| Grenada | 29.97 | 1.91 | 24.62 | 1.57 | 5.35 | 0.34 |
| Guyana | 576.96 | 3.3 | 485.18 | 2.77 | 91.79 | 0.52 |
| Haiti | 1148.2 | 2.85 | 821.52 | 2.04 | 326.68 | 0.81 |
| Jamaica | 334.26 | 1.25 | 275.54 | 1.03 | 58.72 | 0.22 |
| Saint Lucia | 61.02 | 1.8 | 51.47 | 1.52 | 9.55 | 0.28 |
| Saint Vincent and the Grenadines | 35.28 | 1.94 | 28.25 | 1.56 | 7.03 | 0.39 |
| Suriname | 189.11 | 1.77 | 166.03 | 1.55 | 23.08 | 0.22 |
| Trinidad and Tobago | 1109.11 | 2.58 | 901.31 | 2.10 | 207.8 | 0.48 |
| Plurinational State of Bolivia | 812.36 | 0.73 | 682.33 | 0.61 | 130.03 | 0.12 |
| Ecuador | 1794.27 | 0.73 | 1553.19 | 0.63 | 241.08 | 0.10 |
| Peru | 2531.33 | 0.46 | 2210.99 | 0.40 | 320.34 | 0.06 |
| Colombia | 6772.68 | 0.79 | 6174.76 | 0.72 | 597.91 | 0.07 |
| Costa Rica | 1083.97 | 0.96 | 1004.93 | 0.89 | 79.05 | 0.07 |
| El Salvador | 831.28 | 1.19 | 724.05 | 1.04 | 107.23 | 0.15 |
| Guatemala | 3695.85 | 1.98 | 3080.47 | 1.65 | 615.38 | 0.33 |
| Honduras | 714.54 | 1.14 | 665.44 | 1.06 | 49.10 | 0.08 |
| Mexico | 49463.47 | 1.84 | 41036.23 | 1.53 | 8427.24 | 0.31 |
| Nicaragua | 532.10 | 1.13 | 479.43 | 1.01 | 52.67 | 0.11 |
| Panama | 1134.91 | 0.85 | 1006.00 | 0.76 | 128.91 | 0.10 |
| Bolivarian Republic of Venezuela | 1897.32 | 1.16 | 1610.49 | 0.98 | 286.83 | 0.17 |
| Brazil | 30857.59 | 0.77 | 21795.82 | 0.55 | 9061.77 | 0.23 |
| Paraguay | 1057.08 | 0.96 | 908.97 | 0.82 | 148.11 | 0.13 |
| Algeria | 5920.94 | 0.92 | 5200.96 | 0.81 | 719.98 | 0.11 |
| Bahrain | 1463.82 | 1.74 | 1306.14 | 1.55 | 157.68 | 0.19 |
| Egypt | 16838.59 | 1.02 | 13816.25 | 0.84 | 3022.33 | 0.18 |
| Islamic Republic of Iran | 9205.04 | 0.72 | 7783.26 | 0.61 | 1421.78 | 0.11 |
| Iraq | 9245.64 | 1.76 | 8142.29 | 1.55 | 1103.35 | 0.21 |
| Jordan | 1215.08 | 1.07 | 1090.78 | 0.96 | 124.31 | 0.11 |
| Kuwait | 3746.82 | 1.59 | 3420.11 | 1.45 | 326.71 | 0.14 |
| Lebanon | 856.43 | 1.33 | 747.36 | 1.16 | 109.07 | 0.17 |
| Libya | 1051.52 | 1.21 | 891.81 | 1.03 | 159.71 | 0.18 |
| Morocco | 4076.41 | 1.27 | 3650.07 | 1.14 | 426.34 | 0.13 |
| Palestine | 239.46 | 0.82 | 204.35 | 0.7 | 35.11 | 0.12 |
| Oman | 2428.82 | 1.33 | 1814.62 | 1.00 | 614.20 | 0.34 |
| Qatar | 6002.65 | 1.73 | 5426.81 | 1.56 | 575.84 | 0.17 |
| Saudi Arabia | 36916.49 | 1.76 | 32592.86 | 1.55 | 4323.63 | 0.21 |
| Syrian Arab Republic | 433.54 | 0.67 | 359.98 | 0.56 | 73.56 | 0.11 |
| Tunisia | 1253.42 | 0.86 | 1108.30 | 0.76 | 145.12 | 0.10 |
| Turkey | 16015.38 | 0.61 | 13530.76 | 0.52 | 2484.62 | 0.09 |
| United Arab Emirates | 6571.76 | 1.01 | 5672.55 | 0.87 | 899.21 | 0.14 |
| Yemen | 375.84 | 0.59 | 308.36 | 0.48 | 67.47 | 0.11 |
| Afghanistan | 1346.48 | 2.01 | 1090.17 | 1.63 | 256.32 | 0.38 |
| Bangladesh | 15277.46 | 1.25 | 12430.57 | 1.01 | 2846.89 | 0.23 |
| Bhutan | 90.21 | 0.89 | 71.22 | 0.70 | 18.99 | 0.19 |
| India | 117077.34 | 1.03 | 95645.36 | 0.84 | 21431.98 | 0.19 |
| Nepal | 1902.33 | 1.34 | 1612.23 | 1.14 | 290.10 | 0.20 |
| Pakistan | 18620.68 | 1.47 | 14009.39 | 1.11 | 4611.3 | 0.36 |
| Angola | 3466.8 | 1.43 | 2921.79 | 1.21 | 545.00 | 0.22 |
| Central African Republic | 147.31 | 2.38 | 122.13 | 1.97 | 25.18 | 0.41 |
| Congo | 595.62 | 1.76 | 498.02 | 1.47 | 97.6 | 0.29 |
| Democratic Republic of the Congo | 1524.83 | 1.29 | 1236.12 | 1.05 | 288.71 | 0.24 |
| Equatorial Guinea | 484.89 | 1.91 | 415.39 | 1.63 | 69.50 | 0.27 |
| Gabon | 597.77 | 1.78 | 513.14 | 1.53 | 84.62 | 0.25 |
| Burundi | 115.37 | 1.04 | 82.92 | 0.75 | 32.46 | 0.29 |
| Comoros | 31.13 | 1.22 | 24.15 | 0.94 | 6.98 | 0.27 |
| Djibouti | 81.62 | 1.09 | 60.46 | 0.81 | 21.16 | 0.28 |
| Eritrea | 202.76 | 1.61 | 152.03 | 1.21 | 50.73 | 0.40 |
| Ethiopia | 2809.28 | 1.00 | 2048.9 | 0.73 | 760.38 | 0.27 |
| Kenya | 1977.58 | 0.74 | 1440.36 | 0.54 | 537.23 | 0.2 |
| Madagascar | 455.51 | 1.00 | 334.19 | 0.73 | 121.32 | 0.27 |
| Malawi | 378.89 | 1.15 | 265.00 | 0.81 | 113.88 | 0.35 |
| Mauritius | 590.08 | 2.04 | 455.1 | 1.57 | 134.98 | 0.47 |
| Mozambique | 692.30 | 1.53 | 502.13 | 1.11 | 190.16 | 0.42 |
| Rwanda | 328.67 | 0.91 | 227.17 | 0.63 | 101.50 | 0.28 |
| Seychelles | 31.39 | 0.99 | 28.07 | 0.89 | 3.32 | 0.10 |
| Somalia | 420.20 | 1.4 | 293.99 | 0.98 | 126.21 | 0.42 |
| United Republic of Tanzania | 1822.05 | 0.89 | 1275.97 | 0.62 | 546.08 | 0.27 |
| Uganda | 1127.02 | 0.97 | 819.17 | 0.70 | 307.85 | 0.26 |
| Zambia | 1161.82 | 1.70 | 914.92 | 1.34 | 246.9 | 0.36 |
| Botswana | 416.50 | 0.97 | 341.02 | 0.79 | 75.48 | 0.18 |
| Lesotho | 72.75 | 1.53 | 57.11 | 1.20 | 15.64 | 0.33 |
| Namibia | 194.72 | 0.82 | 155.18 | 0.66 | 39.53 | 0.17 |
| South Africa | 10376.05 | 1.33 | 8835.03 | 1.13 | 1541.01 | 0.20 |
| Kingdom of Eswatini | 211.50 | 1.86 | 172.84 | 1.52 | 38.66 | 0.34 |
| Zimbabwe | 524.69 | 1.06 | 436.56 | 0.88 | 88.13 | 0.18 |
| Benin | 558.90 | 1.2 | 500.60 | 1.07 | 58.30 | 0.12 |
| Burkina Faso | 563.39 | 1.00 | 467.57 | 0.83 | 95.82 | 0.17 |
| Cameroon | 2023.28 | 1.33 | 1748.22 | 1.15 | 275.07 | 0.18 |
| Republic of Cabo Verde | 49.51 | 1.15 | 45.63 | 1.06 | 3.88 | 0.09 |
| Chad | 279.72 | 0.93 | 240.75 | 0.8 | 38.97 | 0.13 |
| Republic of C涔坱e d'Ivoire | 2023.08 | 1.20 | 1764.9 | 1.05 | 258.18 | 0.15 |
| Republic of the Gambia | 83.44 | 1.25 | 71.57 | 1.08 | 11.87 | 0.18 |
| Ghana | 2865.58 | 1.27 | 2559.27 | 1.13 | 306.31 | 0.14 |
| Guinea | 530.15 | 1.06 | 450.87 | 0.90 | 79.28 | 0.16 |
| Guinea-Bissau | 89.82 | 1.78 | 76.57 | 1.51 | 13.25 | 0.26 |
| Liberia | 118.98 | 1.42 | 105.2 | 1.25 | 13.78 | 0.16 |
| Mali | 846.5 | 1.5 | 768.44 | 1.36 | 78.05 | 0.14 |
| Mauritania | 156.76 | 0.61 | 131.70 | 0.51 | 25.06 | 0.10 |
| Niger | 287.42 | 0.72 | 249.93 | 0.63 | 37.49 | 0.09 |
| Nigeria | 8905.15 | 0.7 | 7235.82 | 0.57 | 1669.34 | 0.13 |
| Sao Tome and Principe | 11.45 | 0.92 | 10.15 | 0.82 | 1.30 | 0.10 |
| Senegal | 823.26 | 1.24 | 724.15 | 1.09 | 99.11 | 0.15 |
| Sierra Leone | 278.05 | 1.10 | 242.54 | 0.96 | 35.50 | 0.14 |
| Togo | 188.91 | 0.88 | 157.59 | 0.73 | 31.32 | 0.15 |
| American Samoa | 11.82 | 3.39 | 11.48 | 3.3 | 0.33 | 0.10 |
| Bermuda | 31.64 | 0.54 | 26.28 | 0.45 | 5.36 | 0.09 |
| Greenland | 13.11 | 0.34 | 8.83 | 0.23 | 4.29 | 0.11 |
| Guam | 65.45 | 1.08 | 63.21 | 1.04 | 2.24 | 0.04 |
| Principality of Monaco | 27.60 | 0.34 | 21.88 | 0.27 | 5.73 | 0.07 |
| Republic of Nauru | 5.93 | 4.41 | 5.62 | 4.18 | 0.31 | 0.23 |
| Northern Mariana Islands | 14.89 | 1.47 | 14.41 | 1.42 | 0.48 | 0.05 |
| Republic of Palau | 9.32 | 3.29 | 8.93 | 3.15 | 0.40 | 0.14 |
| Puerto Rico | 1500.37 | 1.12 | 1136.35 | 0.85 | 364.03 | 0.27 |
| Saint Kitts and Nevis | 19.55 | 1.24 | 16.60 | 1.05 | 2.95 | 0.19 |
| Republic of San Marino | 6.88 | 0.32 | 5.41 | 0.26 | 1.47 | 0.07 |
| Tuvalu | 1.56 | 2.36 | 1.45 | 2.2 | 0.11 | 0.16 |
| United States Virgin Islands | 50.68 | 1.26 | 39.71 | 0.99 | 10.97 | 0.27 |
| South Sudan | 78.83 | 1.14 | 58.63 | 0.85 | 20.20 | 0.29 |
| Sudan | 1145.08 | 0.81 | 932.60 | 0.66 | 212.47 | 0.15 |
