# Supplementary material for: Estimating the economic burden of diabetes in young adults: A global analysis based on the GBD 2021 and a value of statistical life year framework
Source: Diabet Med. 2026 Feb 13;43(4):e70255. doi: 10.1111/dme.70255 (PMC12982657; doi:10.1111/dme.70255)
Supplement: Supplementary file 2 — Table S2. The projections of the economic burden for diabetes from 2021 to 2050, generated using income elasticity of the VSL at 1.00. [file DME-43-e70255-s003.docx]

**Supplemental Table 2.** The projections of the economic burden for diabetes from 2021 to 2050, generated using income elasticity of the VSL at 1.00.

|  | Overall Diabetes | | Type 2 diabetes | | Type 1 diabetes | |
| --- | --- | --- | --- | --- | --- | --- |
|  | VLW region (millions) | VLW/GDP(%) | VLW region (millions) | VLW/GDP(%) | VLW region (millions) | VLW/GDP(%) |
| 2022 | 1188874.80 | 0.77 | 958726.99 | 0.62 | 243267.00 | 0.16 |
| 2023 | 1208389.72 | 0.78 | 987409.84 | 0.63 | 244776.34 | 0.16 |
| 2024 | 1227266.75 | 0.78 | 1016666.68 | 0.65 | 246213.24 | 0.16 |
| 2025 | 1245367.26 | 0.79 | 1046422.78 | 0.67 | 247577.31 | 0.16 |
| 2026 | 1262495.59 | 0.80 | 1076603.76 | 0.68 | 248853.34 | 0.16 |
| 2027 | 1278473.41 | 0.81 | 1107202.41 | 0.70 | 250006.15 | 0.16 |
| 2028 | 1293314.11 | 0.81 | 1138259.86 | 0.71 | 251030.49 | 0.16 |
| 2029 | 1307046.33 | 0.82 | 1169777.78 | 0.73 | 251936.09 | 0.16 |
| 2030 | 1319693.98 | 0.82 | 1201744.62 | 0.75 | 252740.62 | 0.16 |
| 2031 | 1331183.19 | 0.83 | 1234134.22 | 0.77 | 253455.00 | 0.16 |
| 2032 | 1341329.91 | 0.83 | 1266972.15 | 0.78 | 254051.77 | 0.16 |
| 2033 | 1350313.94 | 0.83 | 1300486.13 | 0.80 | 254534.91 | 0.16 |
| 2034 | 1358249.20 | 0.84 | 1334827.77 | 0.82 | 254917.17 | 0.16 |
| 2035 | 1365206.42 | 0.84 | 1370115.81 | 0.84 | 255216.67 | 0.16 |
| 2036 | 1371205.32 | 0.84 | 1406480.41 | 0.86 | 255439.85 | 0.16 |
| 2037 | 1376285.12 | 0.84 | 1444137.29 | 0.88 | 255576.60 | 0.16 |
| 2038 | 1380761.29 | 0.84 | 1483425.48 | 0.91 | 255640.52 | 0.16 |
| 2039 | 1384910.91 | 0.84 | 1524645.52 | 0.93 | 255650.94 | 0.16 |
| 2040 | 1388961.89 | 0.85 | 1568083.21 | 0.95 | 255632.31 | 0.16 |
| 2041 | 1393016.64 | 0.85 | 1613982.73 | 0.98 | 255597.95 | 0.16 |
| 2042 | 1397066.89 | 0.85 | 1662564.26 | 1.01 | 255539.30 | 0.16 |
| 2043 | 1401107.17 | 0.85 | 1713996.15 | 1.04 | 255455.45 | 0.16 |
| 2044 | 1405129.49 | 0.85 | 1768455.14 | 1.07 | 255345.95 | 0.16 |
| 2045 | 1409160.16 | 0.86 | 1826181.32 | 1.11 | 255216.63 | 0.15 |
| 2046 | 1413210.65 | 0.86 | 1887411.33 | 1.15 | 255075.47 | 0.15 |
| 2047 | 1417262.77 | 0.86 | 1952354.00 | 1.19 | 254913.21 | 0.15 |
| 2048 | 1421324.03 | 0.86 | 2021278.75 | 1.23 | 254731.01 | 0.15 |
| 2049 | 1425415.01 | 0.87 | 2094496.53 | 1.27 | 254531.66 | 0.15 |
| 2050 | 1429575.56 | 0.87 | 2172379.75 | 1.32 | 254322.50 | 0.15 |
