# Supplementary material for: Estimating the economic burden of diabetes in young adults: A global analysis based on the GBD 2021 and a value of statistical life year framework
Source: Diabet Med. 2026 Feb 13;43(4):e70255. doi: 10.1111/dme.70255 (PMC12982657; doi:10.1111/dme.70255)
Supplement: Supplementary file 3 — Table S3. VLW and VLW/GDP by GBD regions in 2021 for diabetes in young adults, generated using income elasticity of the VSL at 0.55. [file DME-43-e70255-s001.docx]

**Supplemental Table 3** VLW and VLW/GDP by GBD regions in 2021 for diabetes in Young Adults, generated using income elasticity of the VSL at 0.55.

|  | Overall Diabetes | | Type 2 diabetes | | Type 1 diabetes | |
| --- | --- | --- | --- | --- | --- | --- |
|  | VLW region (millions) | VLW/GDP(%) | VLW region (millions) | VLW/GDP(%) | VLW region (millions) | VLW/GDP(%) |
| Globa | 2053079.73 | 1.34 | 1671422.31 | 1.09 | 381657.42 | 0.25 |
| High SDI | 397550.37 | 0.66 | 291655.78 | 0.48 | 105894.59 | 0.18 |
| High-middle SDI | 583469.67 | 1.15 | 513207.13 | 1.01 | 70262.55 | 0.14 |
| Low SDI | 101000.25 | 5.33 | 80137.00 | 4.23 | 20863.25 | 1.10 |
| Low-middle SDI | 578442.60 | 2.94 | 469951.65 | 2.39 | 108490.94 | 0.55 |
| Middle SDI | 392616.84 | 1.89 | 316470.75 | 1.52 | 76146.09 | 0.37 |
| Andean Latin America | 10900.57 | 1.20 | 9423.63 | 1.04 | 1476.93 | 0.16 |
| Australasia | 5278.56 | 0.30 | 2806.81 | 0.16 | 2471.75 | 0.14 |
| Caribbean | 18724.87 | 3.10 | 14975.62 | 2.48 | 3749.24 | 0.62 |
| Central Asia | 24154.53 | 1.63 | 17040.59 | 1.15 | 7113.94 | 0.48 |
| Central Europe | 19695.09 | 0.47 | 12785.29 | 0.31 | 6909.81 | 0.17 |
| Central Latin America | 122307.43 | 2.83 | 103338.48 | 2.39 | 18968.95 | 0.44 |
| Central Sub-Saharan Africa | 23630.74 | 5.14 | 19624.45 | 4.27 | 4006.29 | 0.87 |
| East Asia | 424394.53 | 1.46 | 398123.60 | 1.37 | 26270.93 | 0.09 |
| Eastern Europe | 52714.00 | 0.76 | 31915.72 | 0.49 | 20798.28 | 0.30 |
| Eastern Sub-Saharan Africa | 50832.26 | 4.35 | 36946.92 | 3.16 | 13885.34 | 1.19 |
| High-income Asia Pacific | 52868.70 | 0.59 | 47514.14 | 0.53 | 5354.56 | 0.06 |
| High-income North America | 175785.41 | 0.68 | 109420.77 | 0.42 | 66364.63 | 0.28 |
| North Africa and Middle East | 205820.68 | 1.80 | 176694.40 | 1.55 | 29126.28 | 0.26 |
| Oceania | 6518.57 | 9.98 | 5891.79 | 9.02 | 626.78 | 0.96 |
| South Asia | 422315.22 | 3.01 | 341200.60 | 2.43 | 81114.62 | 0.58 |
| Southeast Asia | 147144.82 | 1.65 | 117152.95 | 1.32 | 29991.87 | 0.34 |
| Southern Latin America | 11443.60 | 0.62 | 7990.10 | 0.43 | 3453.51 | 0.19 |
| Southern Sub-Saharan Africa | 26085.37 | 2.86 | 22078.84 | 2.42 | 4006.54 | 0.44 |
| Tropical Latin America | 59504.77 | 1.45 | 42354.75 | 1.04 | 17150.03 | 0.42 |
| Western Europe | 122379.00 | 0.50 | 94155.69 | 0.39 | 28223.32 | 0.12 |
| Western Sub-Saharan Africa | 70581.00 | 3.12 | 59987.17 | 2.65 | 10593.83 | 0.47 |
