# Supplementary material for: Estimating the economic burden of diabetes in young adults: A global analysis based on the GBD 2021 and a value of statistical life year framework
Source: Diabet Med. 2026 Feb 13;43(4):e70255. doi: 10.1111/dme.70255 (PMC12982657; doi:10.1111/dme.70255)
Supplement: Supplementary file 4 — Table S4. VLW and VLW/GDP by GBD regions in 2021 for diabetes in young adults, generated using income elasticity of the VSL at 1.5. [file DME-43-e70255-s004.docx]

**Supplemental Table 4** VLW and VLW/GDP by GBD regions in 2021 for diabetes in Young Adults, generated using income elasticity of the VSL at 1.5

|  | Overall Diabetes | | Type 2 diabetes | | Type 1 diabetes | |
| --- | --- | --- | --- | --- | --- | --- |
|  | VLW region (millions) | VLW/GDP(%) | VLW region (millions) | VLW/GDP(%) | VLW region (millions) | VLW/GDP(%) |
| Globa | 728546.74 | 0.47 | 569325.75 | 0.37 | 159221.00 | 0.10 |
| High SDI | 352037.49 | 0.58 | 255277.05 | 0.42 | 96760.44 | 0.16 |
| High-middle SDI | 207314.34 | 0.41 | 176904.10 | 0.35 | 30410.24 | 0.06 |
| Low SDI | 4635.00 | 0.24 | 3728.69 | 0.20 | 906.32 | 0.05 |
| Low-middle SDI | 70042.89 | 0.36 | 57097.38 | 0.29 | 12945.51 | 0.07 |
| Middle SDI | 94517.03 | 0.46 | 76318.53 | 0.37 | 18198.50 | 0.09 |
| Andean Latin America | 2242.19 | 0.25 | 1942.48 | 0.21 | 299.72 | 0.03 |
| Australasia | 4190.42 | 0.24 | 2197.67 | 0.13 | 1992.76 | 0.11 |
| Caribbean | 5072.79 | 0.84 | 4112.34 | 0.68 | 960.45 | 0.16 |
| Central Asia | 6791.71 | 0.46 | 5018.27 | 0.34 | 1773.44 | 0.12 |
| Central Europe | 10611.37 | 0.25 | 6936.79 | 0.17 | 3674.57 | 0.09 |
| Central Latin America | 34075.93 | 0.79 | 28710.24 | 0.67 | 5365.69 | 0.12 |
| Central Sub-Saharan Africa | 2052.92 | 0.45 | 1730.13 | 0.38 | 322.78 | 0.07 |
| East Asia | 128027.28 | 0.44 | 120150.46 | 0.41 | 7876.83 | 0.03 |
| Eastern Europe | 26708.26 | 0.38 | 16044.19 | 0.23 | 10664.07 | 0.15 |
| Eastern Sub-Saharan Africa | 2380.57 | 0.20 | 1733.62 | 0.15 | 646.94 | 0.06 |
| High-income Asia Pacific | 39688.90 | 0.44 | 35826.27 | 0.40 | 3862.63 | 0.04 |
| High-income North America | 172767.44 | 0.67 | 108105.14 | 0.42 | 64662.30 | 0.25 |
| North Africa and Middle East | 85833.86 | 0.75 | 74573.51 | 0.65 | 11260.35 | 0.10 |
| Oceania | 568.49 | 0.87 | 518.72 | 0.79 | 49.77 | 0.08 |
| South Asia | 49738.38 | 0.36 | 40301.87 | 0.29 | 9436.51 | 0.07 |
| Southeast Asia | 30011.56 | 0.34 | 24189.17 | 0.27 | 5822.39 | 0.07 |
| Southern Latin America | 4545.46 | 0.25 | 3180.56 | 0.17 | 1364.90 | 0.07 |
| Southern Sub-Saharan Africa | 5017.66 | 0.55 | 4255.99 | 0.47 | 761.67 | 0.08 |
| Tropical Latin America | 15975.36 | 0.39 | 11359.17 | 0.28 | 4616.19 | 0.11 |
| Western Europe | 96811.62 | 0.40 | 73832.22 | 0.30 | 22979.40 | 0.10 |
| Western Sub-Saharan Africa | 5434.57 | 0.24 | 4606.93 | 0.20 | 827.64 | 0.04 |
