# Supplementary material for: Estimating the economic burden of diabetes in young adults: A global analysis based on the GBD 2021 and a value of statistical life year framework
Source: Diabet Med. 2026 Feb 13;43(4):e70255. doi: 10.1111/dme.70255 (PMC12982657; doi:10.1111/dme.70255)
Supplement: Supplementary file 5 — Table S5. VLW and VLW/GDP by GBD countries and territories in 2021 for diabetes in young adults, generated using income elasticity of the VSL at 0.55. [file DME-43-e70255-s002.docx]

**Supplemental Table 5** VLW and VLW/GDP by GBD countries and territories in 2021 for diabetes in Young Adults, generated using income elasticity of the VSL at 0.55.

|  | Overall Diabetes | | Type 2 diabetes | | Type 1 diabetes | |
| --- | --- | --- | --- | --- | --- | --- |
|  | VLW region (millions) | VLW/GDP(%) | VLW region (millions) | VLW/GDP(%) | VLW region (millions) | VLW/GDP(%) |
| United States of America | 165793.57 | 0.70 | 106068.50 | 0.45 | 59725.07 | 0.25 |
| China | 422742.95 | 1.46 | 396744.52 | 1.37 | 25998.43 | 0.09 |
| Democratic People's Republic of Korea | 1651.57 | 5.69 | 1379.08 | 4.75 | 272.50 | 0.94 |
| Cambodia | 2568.81 | 2.42 | 2007.23 | 1.89 | 561.58 | 0.53 |
| Indonesia | 55491.00 | 1.56 | 42569.09 | 1.20 | 12921.91 | 0.36 |
| Lao People's Democratic Republic | 1581.99 | 2.65 | 1242.10 | 2.08 | 339.89 | 0.57 |
| Malaysia | 11625.6 | 1.23 | 9947.64 | 1.05 | 1677.96 | 0.18 |
| Maldives | 131.91 | 1.34 | 110.43 | 1.12 | 21.47 | 0.22 |
| Myanmar | 13186.46 | 4.51 | 10290.58 | 3.52 | 2895.88 | 0.99 |
| Philippines | 23651.44 | 2.36 | 18760.82 | 1.87 | 4890.62 | 0.49 |
| Sri Lanka | 6583.99 | 2.07 | 5663.78 | 1.78 | 920.21 | 0.29 |
| Thailand | 16173.29 | 1.20 | 13710.01 | 1.02 | 2463.29 | 0.18 |
| Timor-Leste | 162.54 | 1.70 | 127.78 | 1.34 | 34.76 | 0.36 |
| Socialist Republic of Viet Nam | 14951.47 | 1.24 | 11918.52 | 0.99 | 3032.96 | 0.25 |
| Fiji | 962.22 | 9.74 | 881.37 | 8.92 | 80.85 | 0.82 |
| Kiribati | 67.46 | 18.26 | 64.01 | 17.32 | 3.45 | 0.93 |
| Marshall Islands | 68.46 | 18.14 | 65.56 | 17.37 | 2.90 | 0.77 |
| Federated States of Micronesia | 46.32 | 11.51 | 43.47 | 10.81 | 2.85 | 0.71 |
| Papua New Guinea | 4695.38 | 11.29 | 4205.08 | 10.11 | 490.31 | 1.18 |
| Samoa | 102.93 | 7.75 | 98.40 | 7.41 | 4.53 | 0.34 |
| Solomon Islands | 253.02 | 14.7 | 226.86 | 13.18 | 26.16 | 1.52 |
| Tonga | 41.73 | 5.58 | 39.34 | 5.26 | 2.40 | 0.32 |
| Vanuatu | 97.69 | 9.95 | 90.96 | 9.27 | 6.73 | 0.69 |
| Armenia | 601.04 | 1.26 | 403.94 | 0.85 | 197.09 | 0.41 |
| Azerbaijan | 3002.76 | 1.42 | 1931.76 | 0.91 | 1071.00 | 0.51 |
| Georgia | 922.08 | 1.35 | 644.42 | 0.94 | 277.66 | 0.41 |
| Kazakhstan | 7637.45 | 1.22 | 6205.21 | 0.99 | 1432.24 | 0.23 |
| Kyrgyzstan | 709.33 | 1.8 | 512.81 | 1.30 | 196.52 | 0.50 |
| Mongolia | 725.78 | 1.47 | 542.64 | 1.10 | 183.14 | 0.37 |
| Tajikistan | 1057.32 | 2.61 | 712.09 | 1.76 | 345.23 | 0.85 |
| Turkmenistan | 1895.03 | 2.24 | 1152.85 | 1.36 | 742.19 | 0.88 |
| Uzbekistan | 7603.74 | 2.4 | 4934.87 | 1.56 | 2668.87 | 0.84 |
| Albania | 189.84 | 0.44 | 124.62 | 0.29 | 65.22 | 0.15 |
| Bosnia and Herzegovina | 463.26 | 0.77 | 293.28 | 0.49 | 169.99 | 0.28 |
| Bulgaria | 1533.68 | 0.77 | 882.52 | 0.44 | 651.16 | 0.33 |
| Croatia | 587.63 | 0.38 | 369.42 | 0.24 | 218.21 | 0.14 |
| Czech Republic | 1865.44 | 0.37 | 1195.9 | 0.24 | 669.55 | 0.13 |
| Hungary | 1517.53 | 0.41 | 1030.28 | 0.28 | 487.25 | 0.13 |
| North Macedonia | 423.97 | 0.88 | 233.92 | 0.49 | 190.05 | 0.39 |
| Montenegro | 111.10 | 0.75 | 58.38 | 0.39 | 52.73 | 0.35 |
| Poland | 8281.87 | 0.53 | 5510.83 | 0.35 | 2771.04 | 0.18 |
| Romania | 2199.22 | 0.31 | 1537.52 | 0.22 | 661.70 | 0.09 |
| Serbia | 1424.16 | 0.68 | 843.18 | 0.40 | 580.97 | 0.28 |
| Slovakia | 831.82 | 0.4 | 518.23 | 0.25 | 313.59 | 0.15 |
| Slovenia | 265.57 | 0.28 | 187.20 | 0.20 | 78.37 | 0.08 |
| Belarus | 1843.09 | 0.72 | 966.93 | 0.38 | 876.16 | 0.34 |
| Estonia | 394.54 | 0.68 | 208.31 | 0.36 | 186.23 | 0.32 |
| Latvia | 608.58 | 0.88 | 373.22 | 0.54 | 235.36 | 0.34 |
| Lithuania | 753.53 | 0.60 | 406.71 | 0.32 | 346.82 | 0.28 |
| Republic of Moldova | 890.58 | 1.58 | 582.52 | 1.03 | 308.06 | 0.55 |
| Russian Federation | 40413.97 | 0.72 | 24210.94 | 0.43 | 16203.03 | 0.29 |
| Ukraine | 7809.71 | 1.02 | 5167.09 | 0.67 | 2642.62 | 0.34 |
| Brunei Darussalam | 680.04 | 1.93 | 525.88 | 1.49 | 154.16 | 0.44 |
| Japan | 23447.52 | 0.41 | 20616.38 | 0.36 | 2831.15 | 0.05 |
| Republic of Korea | 24769.78 | 0.99 | 22651.38 | 0.91 | 2118.4 | 0.08 |
| Singapore | 3971.36 | 0.53 | 3720.51 | 0.49 | 250.84 | 0.03 |
| Australia | 4307.48 | 0.29 | 2068.69 | 0.14 | 2238.79 | 0.15 |
| New Zealand | 971.08 | 0.39 | 738.12 | 0.3 | 232.96 | 0.09 |
| Andorra | 24.62 | 0.48 | 18.05 | 0.36 | 6.56 | 0.13 |
| Austria | 1758.58 | 0.31 | 1154.19 | 0.2 | 604.4 | 0.11 |
| Belgium | 3420.09 | 0.49 | 2751.2 | 0.4 | 668.9 | 0.1 |
| Cyprus | 464.76 | 0.70 | 317.4 | 0.48 | 147.36 | 0.22 |
| Denmark | 1312.91 | 0.32 | 878.95 | 0.22 | 433.95 | 0.11 |
| Finland | 2294.08 | 0.73 | 1387.89 | 0.44 | 906.18 | 0.29 |
| France | 11307.14 | 0.32 | 8025.61 | 0.23 | 3281.53 | 0.09 |
| Germany | 22242.19 | 0.42 | 16393.75 | 0.31 | 5848.44 | 0.11 |
| Greece | 2370.73 | 0.69 | 2054.72 | 0.60 | 316.01 | 0.09 |
| Iceland | 103.42 | 0.48 | 83.39 | 0.39 | 20.03 | 0.09 |
| Ireland | 1700.43 | 0.29 | 1075.05 | 0.18 | 625.38 | 0.11 |
| Israel | 1945.34 | 0.44 | 1469.48 | 0.33 | 475.86 | 0.11 |
| Italy | 10336.59 | 0.35 | 6126.49 | 0.21 | 4210.09 | 0.14 |
| Luxembourg | 301.26 | 0.35 | 242.19 | 0.28 | 59.06 | 0.07 |
| Malta | 158.25 | 0.63 | 116.15 | 0.46 | 42.10 | 0.17 |
| Netherlands | 4209.39 | 0.36 | 2913.9 | 0.25 | 1295.49 | 0.11 |
| Norway | 2070.49 | 0.43 | 1302.14 | 0.27 | 768.35 | 0.16 |
| Portugal | 2939.73 | 0.72 | 2505.91 | 0.61 | 433.82 | 0.11 |
| Spain | 11847.38 | 0.59 | 9570.68 | 0.47 | 2276.70 | 0.11 |
| Sweden | 2992.67 | 0.46 | 1981.10 | 0.30 | 1011.57 | 0.16 |
| Switzerland | 3760.6 | 0.52 | 3210.36 | 0.45 | 550.25 | 0.08 |
| United Kingdom of Great Britain and Northern Ireland | 34794.29 | 1.01 | 30558.05 | 0.88 | 4236.24 | 0.12 |
| Argentina | 8014.31 | 0.67 | 5420.07 | 0.45 | 2594.25 | 0.22 |
| Chile | 2863.22 | 0.52 | 2173.01 | 0.40 | 690.22 | 0.13 |
| Uruguay | 566.07 | 0.56 | 397.02 | 0.40 | 169.04 | 0.17 |
| Canada | 9978.41 | 0.47 | 3343.24 | 0.16 | 6635.17 | 0.31 |
| Antigua and Barbuda | 45.23 | 1.97 | 35.32 | 1.54 | 9.91 | 0.43 |
| Commonwealth of the Bahamas | 302.3 | 2.66 | 237.73 | 2.09 | 64.56 | 0.57 |
| Barbados | 114.04 | 2.43 | 87.96 | 1.88 | 26.08 | 0.56 |
| Belize | 172.86 | 3.45 | 137.74 | 2.75 | 35.12 | 0.70 |
| Cuba | 1140.87 | 2.37 | 1029.42 | 2.14 | 111.45 | 0.23 |
| Dominica | 36.82 | 3.52 | 30.66 | 2.93 | 6.16 | 0.59 |
| Dominican Republic | 6151.3 | 2.55 | 5172.54 | 2.14 | 978.76 | 0.41 |
| Grenada | 60.09 | 3.83 | 49.37 | 3.14 | 10.72 | 0.68 |
| Guyana | 965.37 | 5.52 | 811.8 | 4.64 | 153.58 | 0.88 |
| Haiti | 4697.83 | 11.65 | 3361.24 | 8.33 | 1336.59 | 3.31 |
| Jamaica | 827.39 | 3.09 | 682.05 | 2.54 | 145.34 | 0.54 |
| Saint Lucia | 110.71 | 3.27 | 93.37 | 2.75 | 17.33 | 0.51 |
| Saint Vincent and the Grenadines | 69.51 | 3.83 | 55.65 | 3.07 | 13.85 | 0.76 |
| Suriname | 348.43 | 3.26 | 305.91 | 2.86 | 42.52 | 0.40 |
| Trinidad and Tobago | 1622.35 | 3.78 | 1318.39 | 3.07 | 303.96 | 0.71 |
| Plurinational State of Bolivia | 2022.56 | 1.81 | 1698.82 | 1.52 | 323.74 | 0.29 |
| Ecuador | 3800.55 | 1.55 | 3289.9 | 1.35 | 510.64 | 0.21 |
| Peru | 5077.46 | 0.92 | 4434.91 | 0.8 | 642.55 | 0.12 |
| Colombia | 12819.95 | 1.50 | 11688.16 | 1.37 | 1131.79 | 0.13 |
| Costa Rica | 1779.54 | 1.57 | 1649.77 | 1.46 | 129.77 | 0.11 |
| El Salvador | 1948.57 | 2.79 | 1697.22 | 2.43 | 251.36 | 0.36 |
| Guatemala | 8319.22 | 4.46 | 6934.02 | 3.72 | 1385.2 | 0.74 |
| Honduras | 2150.50 | 3.43 | 2002.74 | 3.19 | 147.76 | 0.24 |
| Mexico | 86397.84 | 3.22 | 71677.97 | 2.67 | 14719.87 | 0.55 |
| Nicaragua | 1508.27 | 3.19 | 1358.98 | 2.88 | 149.29 | 0.32 |
| Panama | 1657.51 | 1.25 | 1469.24 | 1.11 | 188.27 | 0.14 |
| Bolivarian Republic of Venezuela | 5726.04 | 3.49 | 4860.39 | 2.96 | 865.65 | 0.53 |
| Brazil | 57392.12 | 1.44 | 40538.11 | 1.02 | 16854.01 | 0.42 |
| Paraguay | 2112.65 | 1.91 | 1816.64 | 1.64 | 296.01 | 0.27 |
| Algeria | 12161.87 | 1.9 | 10683.01 | 1.67 | 1478.86 | 0.23 |
| Bahrain | 1650.7 | 1.96 | 1472.89 | 1.75 | 177.81 | 0.21 |
| Egypt | 33484.65 | 2.03 | 27474.54 | 1.67 | 6010.11 | 0.37 |
| Islamic Republic of Iran | 18646.42 | 1.46 | 15766.36 | 1.24 | 2880.06 | 0.23 |
| Iraq | 20133.55 | 3.84 | 17730.87 | 3.38 | 2402.68 | 0.46 |
| Jordan | 3065.18 | 2.71 | 2751.61 | 2.43 | 313.58 | 0.28 |
| Kuwait | 4381.61 | 1.86 | 3999.55 | 1.7 | 382.06 | 0.16 |
| Lebanon | 1944.79 | 3.03 | 1697.11 | 2.64 | 247.68 | 0.39 |
| Libya | 2297.17 | 2.64 | 1948.26 | 2.24 | 348.91 | 0.40 |
| Morocco | 10578.12 | 3.30 | 9471.79 | 2.95 | 1106.33 | 0.35 |
| Palestine | 750.84 | 2.58 | 640.74 | 2.20 | 110.10 | 0.38 |
| Oman | 3206.34 | 1.76 | 2395.52 | 1.32 | 810.82 | 0.45 |
| Qatar | 4820.83 | 1.39 | 4358.37 | 1.25 | 462.47 | 0.13 |
| Saudi Arabia | 41355.13 | 1.97 | 36511.65 | 1.74 | 4843.48 | 0.23 |
| Syrian Arab Republic | 1493.74 | 2.32 | 1240.29 | 1.92 | 253.45 | 0.39 |
| Tunisia | 2764.59 | 1.89 | 2444.50 | 1.67 | 320.08 | 0.22 |
| Turkey | 23253.41 | 0.89 | 19645.89 | 0.75 | 3607.52 | 0.14 |
| United Arab Emirates | 6760.28 | 1.04 | 5835.27 | 0.90 | 925.00 | 0.14 |
| Yemen | 1925.02 | 3.01 | 1579.41 | 2.47 | 345.6 | 0.54 |
| Afghanistan | 6536.09 | 9.76 | 5291.88 | 7.9 | 1244.21 | 1.86 |
| Bangladesh | 42364.13 | 3.46 | 34469.75 | 2.81 | 7894.37 | 0.64 |
| Bhutan | 191.60 | 1.88 | 151.26 | 1.48 | 40.33 | 0.40 |
| India | 313362.77 | 2.75 | 255999.11 | 2.25 | 57363.66 | 0.50 |
| Nepal | 6584.56 | 4.65 | 5580.42 | 3.94 | 1004.14 | 0.71 |
| Pakistan | 59812.17 | 4.73 | 45000.06 | 3.56 | 14812.11 | 1.17 |
| Angola | 9632.59 | 3.98 | 8118.28 | 3.35 | 1514.31 | 0.62 |
| Central African Republic | 954.49 | 15.42 | 791.33 | 12.79 | 163.15 | 2.64 |
| Congo | 1784.83 | 5.29 | 1492.35 | 4.42 | 292.48 | 0.87 |
| Democratic Republic of the Congo | 9227.72 | 7.80 | 7480.56 | 6.33 | 1747.16 | 1.48 |
| Equatorial Guinea | 931.51 | 3.66 | 797.99 | 3.14 | 133.52 | 0.52 |
| Gabon | 1099.61 | 3.27 | 943.94 | 2.81 | 155.67 | 0.46 |
| Burundi | 855.34 | 7.73 | 614.73 | 5.56 | 240.61 | 2.18 |
| Comoros | 122.13 | 4.77 | 94.73 | 3.70 | 27.40 | 1.07 |
| Djibouti | 250.33 | 3.34 | 185.44 | 2.48 | 64.89 | 0.87 |
| Eritrea | 1036.79 | 8.23 | 777.39 | 6.17 | 259.40 | 2.06 |
| Ethiopia | 12528.90 | 4.44 | 9137.72 | 3.24 | 3391.18 | 1.20 |
| Kenya | 6367.46 | 2.38 | 4637.70 | 1.74 | 1729.77 | 0.65 |
| Madagascar | 2522.91 | 5.52 | 1850.96 | 4.05 | 671.95 | 1.47 |
| Malawi | 2048.43 | 6.24 | 1432.73 | 4.37 | 615.70 | 1.88 |
| Mauritius | 989.83 | 3.42 | 763.40 | 2.64 | 226.43 | 0.78 |
| Mozambique | 3998.41 | 8.83 | 2900.10 | 6.40 | 1098.31 | 2.43 |
| Rwanda | 1430.84 | 3.95 | 988.98 | 2.73 | 441.86 | 1.22 |
| Seychelles | 46.49 | 1.47 | 41.58 | 1.32 | 4.91 | 0.16 |
| Somalia | 2477.07 | 8.23 | 1733.04 | 5.76 | 744.03 | 2.47 |
| United Republic of Tanzania | 7100.67 | 3.48 | 4972.56 | 2.44 | 2128.11 | 1.04 |
| Uganda | 4944.21 | 4.25 | 3593.69 | 3.09 | 1350.52 | 1.16 |
| Zambia | 4521.89 | 6.61 | 3560.94 | 5.21 | 960.95 | 1.41 |
| Botswana | 776.86 | 1.81 | 636.07 | 1.48 | 140.79 | 0.33 |
| Lesotho | 326.95 | 6.85 | 256.65 | 5.38 | 70.31 | 1.47 |
| Namibia | 479.02 | 2.03 | 381.77 | 1.62 | 97.25 | 0.41 |
| South Africa | 21853.94 | 2.8 | 18608.27 | 2.39 | 3245.67 | 0.42 |
| Kingdom of Eswatini | 517.03 | 4.55 | 422.53 | 3.71 | 94.50 | 0.83 |
| Zimbabwe | 2131.56 | 4.29 | 1773.55 | 3.57 | 358.02 | 0.72 |
| Benin | 2186.21 | 4.67 | 1958.15 | 4.19 | 228.05 | 0.49 |
| Burkina Faso | 2558.48 | 4.52 | 2123.32 | 3.75 | 435.16 | 0.77 |
| Cameroon | 6838.24 | 4.49 | 5908.58 | 3.88 | 929.66 | 0.61 |
| Republic of Cabo Verde | 135.31 | 3.15 | 124.70 | 2.90 | 10.61 | 0.25 |
| Chad | 1507.26 | 4.99 | 1297.29 | 4.30 | 209.97 | 0.70 |
| Republic of C涔坱e d'Ivoire | 6159.73 | 3.66 | 5373.63 | 3.19 | 786.1 | 0.47 |
| Republic of the Gambia | 360.51 | 5.42 | 309.22 | 4.65 | 51.28 | 0.77 |
| Ghana | 8382.61 | 3.70 | 7486.56 | 3.31 | 896.05 | 0.40 |
| Guinea | 2003.81 | 3.99 | 1704.14 | 3.39 | 299.67 | 0.60 |
| Guinea-Bissau | 410.54 | 8.12 | 349.97 | 6.92 | 60.58 | 1.20 |
| Liberia | 670.49 | 7.98 | 592.83 | 7.05 | 77.66 | 0.92 |
| Mali | 3944.80 | 6.97 | 3581.06 | 6.33 | 363.74 | 0.64 |
| Mauritania | 484.90 | 1.89 | 407.37 | 1.59 | 77.53 | 0.30 |
| Niger | 1598.13 | 4.03 | 1389.66 | 3.50 | 208.48 | 0.53 |
| Nigeria | 28311.08 | 2.23 | 23003.96 | 1.81 | 5307.12 | 0.42 |
| Sao Tome and Principe | 35.71 | 2.88 | 31.66 | 2.55 | 4.05 | 0.33 |
| Senegal | 2961.34 | 4.47 | 2604.83 | 3.94 | 356.51 | 0.54 |
| Sierra Leone | 1187.71 | 4.70 | 1036.05 | 4.10 | 151.65 | 0.60 |
| Togo | 844.14 | 3.91 | 704.18 | 3.26 | 139.97 | 0.65 |
| American Samoa | 33.68 | 9.67 | 32.73 | 9.40 | 0.95 | 0.27 |
| Bermuda | 28.26 | 0.48 | 23.47 | 0.40 | 4.79 | 0.08 |
| Greenland | 13.43 | 0.35 | 9.04 | 0.24 | 4.39 | 0.11 |
| Guam | 87.05 | 1.44 | 84.07 | 1.39 | 2.98 | 0.05 |
| Principality of Monaco | 16.88 | 0.21 | 13.38 | 0.17 | 3.50 | 0.04 |
| Republic of Nauru | 13.17 | 9.79 | 12.47 | 9.28 | 0.69 | 0.52 |
| Northern Mariana Islands | 25.92 | 2.55 | 25.08 | 2.47 | 0.84 | 0.08 |
| Republic of Palau | 18.5 | 6.53 | 17.71 | 6.25 | 0.79 | 0.28 |
| Puerto Rico | 1939.72 | 1.45 | 1469.1 | 1.10 | 470.63 | 0.35 |
| Saint Kitts and Nevis | 30.41 | 1.93 | 25.82 | 1.64 | 4.59 | 0.29 |
| Republic of San Marino | 7.21 | 0.34 | 5.67 | 0.27 | 1.54 | 0.07 |
| Tuvalu | 5.02 | 7.59 | 4.68 | 7.07 | 0.34 | 0.52 |
| United States Virgin Islands | 61.38 | 1.52 | 48.09 | 1.19 | 13.29 | 0.33 |
| South Sudan | 626.87 | 9.05 | 466.21 | 6.73 | 160.66 | 2.32 |
| Sudan | 4610.34 | 3.27 | 3754.87 | 2.66 | 855.47 | 0.61 |
