# Supplementary material for: Estimating the economic burden of diabetes in young adults: A global analysis based on the GBD 2021 and a value of statistical life year framework
Source: Diabet Med. 2026 Feb 13;43(4):e70255. doi: 10.1111/dme.70255 (PMC12982657; doi:10.1111/dme.70255)
Supplement: Supplementary file 6 — Table S6. VLW and VLW/GDP by GBD countries and territories in 2021 for diabetes in young adults, generated using income elasticity of the VSL at 1.5. [file DME-43-e70255-s006.docx]

**Supplemental Table 6** VLW and VLW/GDP by GBD countries and territories in 2021 for diabetes in Young Adults, generated using income elasticity of the VSL at 1.5.

|  | Overall Diabetes | | Type 1 diabetes | | Type 2 diabetes | |
| --- | --- | --- | --- | --- | --- | --- |
|  | VLW region (millions) | VLW/GDP(%) | VLW region (millions) | VLW/GDP(%) | VLW region (millions) | VLW/GDP(%) |
| United States of America | 164795.08 | 0.69 | 59365.38 | 0.25 | 105429.7 | 0.44 |
| China | 127996.09 | 0.44 | 7871.68 | 0.03 | 120124.41 | 0.41 |
| Democratic People's Republic of Korea | 31.19 | 0.11 | 5.15 | 0.02 | 26.05 | 0.09 |
| Cambodia | 251.79 | 0.24 | 55.04 | 0.05 | 196.74 | 0.19 |
| Indonesia | 10752.8 | 0.30 | 2503.95 | 0.07 | 8248.85 | 0.23 |
| Lao People's Democratic Republic | 198.66 | 0.33 | 42.68 | 0.07 | 155.97 | 0.26 |
| Malaysia | 5047.46 | 0.53 | 728.52 | 0.08 | 4318.94 | 0.46 |
| Maldives | 37.42 | 0.38 | 6.09 | 0.06 | 31.33 | 0.32 |
| Myanmar | 1085.06 | 0.37 | 238.29 | 0.08 | 846.77 | 0.29 |
| Philippines | 3240.8 | 0.32 | 670.13 | 0.07 | 2570.67 | 0.26 |
| Sri Lanka | 1423.51 | 0.45 | 198.96 | 0.06 | 1224.55 | 0.38 |
| Thailand | 4860.05 | 0.36 | 740.21 | 0.05 | 4119.84 | 0.31 |
| Timor-Leste | 17.39 | 0.18 | 3.72 | 0.04 | 13.67 | 0.14 |
| Socialist Republic of Viet Nam | 2744.22 | 0.23 | 556.68 | 0.05 | 2187.55 | 0.18 |
| Fiji | 157.56 | 1.60 | 13.24 | 0.13 | 144.32 | 1.46 |
| Kiribati | 3.36 | 0.91 | 0.17 | 0.05 | 3.18 | 0.86 |
| Marshall Islands | 7.20 | 1.91 | 0.31 | 0.08 | 6.9 | 1.83 |
| Federated States of Micronesia | 2.93 | 0.73 | 0.18 | 0.04 | 2.75 | 0.68 |
| Papua New Guinea | 300.66 | 0.72 | 31.4 | 0.08 | 269.26 | 0.65 |
| Samoa | 10.07 | 0.76 | 0.44 | 0.03 | 9.63 | 0.73 |
| Solomon Islands | 10.50 | 0.61 | 1.09 | 0.06 | 9.41 | 0.55 |
| Tonga | 4.59 | 0.61 | 0.26 | 0.04 | 4.33 | 0.58 |
| Vanuatu | 4.99 | 0.51 | 0.34 | 0.04 | 4.65 | 0.47 |
| Armenia | 143.76 | 0.30 | 47.14 | 0.1 | 96.62 | 0.20 |
| Azerbaijan | 896.66 | 0.42 | 319.81 | 0.15 | 576.84 | 0.27 |
| Georgia | 259.77 | 0.38 | 78.22 | 0.11 | 181.54 | 0.27 |
| Kazakhstan | 3644.98 | 0.58 | 683.54 | 0.11 | 2961.44 | 0.47 |
| Kyrgyzstan | 64.33 | 0.16 | 17.82 | 0.05 | 46.51 | 0.12 |
| Mongolia | 161.87 | 0.33 | 40.85 | 0.08 | 121.03 | 0.25 |
| Tajikistan | 67.85 | 0.17 | 22.15 | 0.05 | 45.70 | 0.11 |
| Turkmenistan | 467.10 | 0.55 | 182.94 | 0.22 | 284.16 | 0.34 |
| Uzbekistan | 1085.41 | 0.34 | 380.97 | 0.12 | 704.44 | 0.22 |
| Albania | 45.97 | 0.11 | 15.79 | 0.04 | 30.17 | 0.07 |
| Bosnia and Herzegovina | 126.39 | 0.21 | 46.37 | 0.08 | 80.01 | 0.13 |
| Bulgaria | 654.89 | 0.33 | 278.05 | 0.14 | 376.84 | 0.19 |
| Croatia | 312.59 | 0.20 | 116.08 | 0.07 | 196.51 | 0.13 |
| Czech Republic | 1267.77 | 0.25 | 455.03 | 0.09 | 812.74 | 0.16 |
| Hungary | 836.13 | 0.23 | 268.46 | 0.07 | 567.66 | 0.15 |
| North Macedonia | 138.73 | 0.29 | 62.19 | 0.13 | 76.54 | 0.16 |
| Montenegro | 39.35 | 0.26 | 18.67 | 0.13 | 20.68 | 0.14 |
| Poland | 4872.00 | 0.31 | 1630.13 | 0.10 | 3241.87 | 0.21 |
| Romania | 1193.95 | 0.17 | 359.23 | 0.05 | 834.71 | 0.12 |
| Serbia | 491.20 | 0.24 | 200.38 | 0.10 | 290.82 | 0.14 |
| Slovakia | 458.68 | 0.22 | 172.92 | 0.08 | 285.76 | 0.14 |
| Slovenia | 173.73 | 0.18 | 51.27 | 0.05 | 122.46 | 0.13 |
| Belarus | 743.73 | 0.29 | 353.55 | 0.14 | 390.18 | 0.15 |
| Estonia | 249.20 | 0.43 | 117.63 | 0.20 | 131.57 | 0.23 |
| Latvia | 323.58 | 0.47 | 125.14 | 0.18 | 198.44 | 0.29 |
| Lithuania | 493.77 | 0.39 | 227.27 | 0.18 | 266.51 | 0.21 |
| Republic of Moldova | 209.96 | 0.37 | 72.63 | 0.13 | 137.34 | 0.24 |
| Russian Federation | 22606.19 | 0.40 | 9063.42 | 0.16 | 13542.77 | 0.24 |
| Ukraine | 2081.82 | 0.27 | 704.44 | 0.09 | 1377.38 | 0.18 |
| Brunei Darussalam | 738.20 | 2.09 | 167.35 | 0.47 | 570.85 | 1.62 |
| Japan | 14830.66 | 0.26 | 1790.71 | 0.03 | 13039.94 | 0.23 |
| Republic of Korea | 17042.30 | 0.68 | 1457.52 | 0.06 | 15584.78 | 0.62 |
| Singapore | 7077.74 | 0.94 | 447.05 | 0.06 | 6630.69 | 0.88 |
| Australia | 3528.67 | 0.24 | 1834.00 | 0.12 | 1694.66 | 0.11 |
| New Zealand | 661.76 | 0.27 | 158.76 | 0.06 | 503.00 | 0.20 |
| Andorra | 20.55 | 0.40 | 5.48 | 0.11 | 15.07 | 0.30 |
| Austria | 1552.99 | 0.27 | 533.74 | 0.09 | 1019.25 | 0.18 |
| Belgium | 2915.24 | 0.42 | 570.16 | 0.08 | 2345.08 | 0.34 |
| Cyprus | 322.06 | 0.49 | 102.12 | 0.15 | 219.94 | 0.33 |
| Denmark | 1277.12 | 0.31 | 422.12 | 0.10 | 854.99 | 0.21 |
| Finland | 1836.56 | 0.58 | 725.46 | 0.23 | 1111.10 | 0.35 |
| France | 8403.80 | 0.24 | 2438.93 | 0.07 | 5964.87 | 0.17 |
| Germany | 19512.02 | 0.37 | 5130.56 | 0.10 | 14381.45 | 0.27 |
| Greece | 1150.52 | 0.34 | 153.36 | 0.04 | 997.16 | 0.29 |
| Iceland | 89.64 | 0.41 | 17.36 | 0.08 | 72.28 | 0.33 |
| Ireland | 2742.39 | 0.47 | 1008.58 | 0.17 | 1733.8 | 0.30 |
| Israel | 1278.74 | 0.29 | 312.80 | 0.07 | 965.94 | 0.22 |
| Italy | 7307.87 | 0.25 | 2976.5 | 0.10 | 4331.37 | 0.15 |
| Luxembourg | 550.96 | 0.63 | 108.02 | 0.12 | 442.94 | 0.51 |
| Malta | 126.60 | 0.50 | 33.68 | 0.13 | 92.92 | 0.37 |
| Netherlands | 4030.92 | 0.34 | 1240.56 | 0.11 | 2790.36 | 0.24 |
| Norway | 2539.54 | 0.53 | 942.41 | 0.20 | 1597.13 | 0.33 |
| Portugal | 1633.11 | 0.40 | 241.00 | 0.06 | 1392.11 | 0.34 |
| Spain | 7487.47 | 0.37 | 1438.86 | 0.07 | 6048.61 | 0.30 |
| Sweden | 2633.34 | 0.40 | 890.11 | 0.14 | 1743.23 | 0.27 |
| Switzerland | 4209.19 | 0.58 | 615.88 | 0.09 | 3593.31 | 0.50 |
| United Kingdom of Great Britain and Northern Ireland | 25136.84 | 0.73 | 3060.44 | 0.09 | 22076.4 | 0.64 |
| Argentina | 3087.91 | 0.26 | 999.56 | 0.08 | 2088.35 | 0.17 |
| Chile | 1214.77 | 0.22 | 292.84 | 0.05 | 921.93 | 0.17 |
| Uruguay | 242.78 | 0.24 | 72.50 | 0.07 | 170.28 | 0.17 |
| Canada | 7959.59 | 0.38 | 5292.75 | 0.25 | 2666.84 | 0.13 |
| Antigua and Barbuda | 17.07 | 0.74 | 3.74 | 0.16 | 13.33 | 0.58 |
| Commonwealth of the Bahamas | 129.23 | 1.14 | 27.60 | 0.24 | 101.63 | 0.89 |
| Barbados | 26.89 | 0.57 | 6.15 | 0.13 | 20.74 | 0.44 |
| Belize | 30.80 | 0.61 | 6.26 | 0.12 | 24.54 | 0.49 |
| Cuba | 78.30 | 0.16 | 7.65 | 0.02 | 70.65 | 0.15 |
| Dominica | 8.63 | 0.83 | 1.44 | 0.14 | 7.19 | 0.69 |
| Dominican Republic | 1992.76 | 0.83 | 317.08 | 0.13 | 1675.69 | 0.69 |
| Grenada | 13.84 | 0.88 | 2.47 | 0.16 | 11.37 | 0.72 |
| Guyana | 325.66 | 1.86 | 51.81 | 0.3 | 273.85 | 1.57 |
| Haiti | 239.97 | 0.6 | 68.27 | 0.17 | 171.69 | 0.43 |
| Jamaica | 122.10 | 0.46 | 21.45 | 0.08 | 100.65 | 0.38 |
| Saint Lucia | 31.48 | 0.93 | 4.93 | 0.15 | 26.55 | 0.78 |
| Saint Vincent and the Grenadines | 16.60 | 0.92 | 3.31 | 0.18 | 13.30 | 0.73 |
| Suriname | 95.90 | 0.90 | 11.70 | 0.11 | 84.20 | 0.79 |
| Trinidad and Tobago | 726.87 | 1.69 | 136.19 | 0.32 | 590.68 | 1.38 |
| Plurinational State of Bolivia | 294.84 | 0.26 | 47.19 | 0.04 | 247.64 | 0.22 |
| Ecuador | 779.31 | 0.32 | 104.71 | 0.04 | 674.60 | 0.28 |
| Peru | 1168.05 | 0.21 | 147.82 | 0.03 | 1020.23 | 0.18 |
| Colombia | 3333.05 | 0.39 | 294.25 | 0.03 | 3038.8 | 0.36 |
| Costa Rica | 624.9 | 0.55 | 45.57 | 0.04 | 579.33 | 0.51 |
| El Salvador | 322.61 | 0.46 | 41.61 | 0.06 | 280.99 | 0.40 |
| Guatemala | 1500.36 | 0.80 | 249.82 | 0.13 | 1250.54 | 0.67 |
| Honduras | 210.06 | 0.33 | 14.43 | 0.02 | 195.63 | 0.31 |
| Mexico | 26616.63 | 0.99 | 4534.76 | 0.17 | 22081.88 | 0.82 |
| Nicaragua | 167.20 | 0.35 | 16.55 | 0.04 | 150.65 | 0.32 |
| Panama | 745.06 | 0.56 | 84.63 | 0.06 | 660.43 | 0.50 |
| Bolivarian Republic of Venezuela | 556.07 | 0.34 | 84.06 | 0.05 | 472.00 | 0.29 |
| Brazil | 15485.61 | 0.39 | 4547.57 | 0.11 | 10938.04 | 0.27 |
| Paraguay | 489.75 | 0.44 | 68.62 | 0.06 | 421.13 | 0.38 |
| Algeria | 2661.01 | 0.42 | 323.57 | 0.05 | 2337.44 | 0.36 |
| Bahrain | 1280.88 | 1.52 | 137.98 | 0.16 | 1142.91 | 1.36 |
| Egypt | 7845.03 | 0.48 | 1408.09 | 0.09 | 6436.93 | 0.39 |
| Islamic Republic of Iran | 4201.38 | 0.33 | 648.93 | 0.05 | 3552.45 | 0.28 |
| Iraq | 3894.03 | 0.74 | 464.70 | 0.09 | 3429.33 | 0.65 |
| Jordan | 434.62 | 0.38 | 44.46 | 0.04 | 390.15 | 0.34 |
| Kuwait | 3148.75 | 1.34 | 274.56 | 0.12 | 2874.19 | 1.22 |
| Lebanon | 344.30 | 0.54 | 43.85 | 0.07 | 300.45 | 0.47 |
| Libya | 441.30 | 0.51 | 67.03 | 0.08 | 374.28 | 0.43 |
| Morocco | 1412.97 | 0.44 | 147.78 | 0.05 | 1265.19 | 0.39 |
| Palestine | 67.26 | 0.23 | 9.86 | 0.03 | 57.40 | 0.20 |
| Oman | 1783.93 | 0.98 | 451.12 | 0.25 | 1332.81 | 0.73 |
| Qatar | 7658.5 | 2.20 | 734.69 | 0.21 | 6923.81 | 1.99 |
| Saudi Arabia | 32541.12 | 1.55 | 3811.19 | 0.18 | 28729.93 | 1.37 |
| Syrian Arab Republic | 109.67 | 0.17 | 18.61 | 0.03 | 91.06 | 0.14 |
| Tunisia | 520.47 | 0.36 | 60.26 | 0.04 | 460.21 | 0.31 |
| Turkey | 10582.63 | 0.40 | 1641.78 | 0.06 | 8940.84 | 0.34 |
| United Arab Emirates | 6368.45 | 0.98 | 871.39 | 0.13 | 5497.06 | 0.85 |
| Yemen | 61.20 | 0.10 | 10.99 | 0.02 | 50.21 | 0.08 |
| Afghanistan | 232.73 | 0.35 | 44.30 | 0.07 | 188.43 | 0.28 |
| Bangladesh | 4919.12 | 0.40 | 916.66 | 0.07 | 4002.46 | 0.33 |
| Bhutan | 39.06 | 0.38 | 8.22 | 0.08 | 30.84 | 0.30 |
| India | 39209.38 | 0.34 | 7177.6 | 0.06 | 32031.78 | 0.28 |
| Nepal | 478.77 | 0.34 | 73.01 | 0.05 | 405.76 | 0.29 |
| Pakistan | 5092.03 | 0.40 | 1261.01 | 0.10 | 3831.02 | 0.30 |
| Angola | 1113.79 | 0.46 | 175.09 | 0.07 | 938.69 | 0.39 |
| Central African Republic | 18.47 | 0.30 | 3.16 | 0.05 | 15.31 | 0.25 |
| Congo | 175.95 | 0.52 | 28.83 | 0.09 | 147.12 | 0.44 |
| Democratic Republic of the Congo | 206.29 | 0.17 | 39.06 | 0.03 | 167.23 | 0.14 |
| Equatorial Guinea | 234.74 | 0.92 | 33.65 | 0.13 | 201.1 | 0.79 |
| Gabon | 303.68 | 0.90 | 42.99 | 0.13 | 260.69 | 0.77 |
| Burundi | 12.46 | 0.11 | 3.50 | 0.03 | 8.95 | 0.08 |
| Comoros | 6.82 | 0.27 | 1.53 | 0.06 | 5.29 | 0.21 |
| Djibouti | 23.50 | 0.31 | 6.09 | 0.08 | 17.41 | 0.23 |
| Eritrea | 33.08 | 0.26 | 8.28 | 0.07 | 24.80 | 0.20 |
| Ethiopia | 533.5 | 0.19 | 144.4 | 0.05 | 389.09 | 0.14 |
| Kenya | 539.36 | 0.2 | 146.52 | 0.05 | 392.84 | 0.15 |
| Madagascar | 68.00 | 0.15 | 18.11 | 0.04 | 49.89 | 0.11 |
| Malawi | 58.10 | 0.18 | 17.46 | 0.05 | 40.64 | 0.12 |
| Mauritius | 332.12 | 1.15 | 75.98 | 0.26 | 256.15 | 0.89 |
| Mozambique | 98.65 | 0.22 | 27.10 | 0.06 | 71.55 | 0.16 |
| Rwanda | 64.11 | 0.18 | 19.8 | 0.05 | 44.31 | 0.12 |
| Seychelles | 20.29 | 0.64 | 2.14 | 0.07 | 18.14 | 0.57 |
| Somalia | 58.53 | 0.19 | 17.58 | 0.06 | 40.95 | 0.14 |
| United Republic of Tanzania | 401.96 | 0.20 | 120.47 | 0.06 | 281.49 | 0.14 |
| Uganda | 217.98 | 0.19 | 59.54 | 0.05 | 158.44 | 0.14 |
| Zambia | 256.67 | 0.38 | 54.55 | 0.08 | 202.13 | 0.30 |
| Botswana | 208.35 | 0.48 | 37.76 | 0.09 | 170.59 | 0.40 |
| Lesotho | 13.70 | 0.29 | 2.95 | 0.06 | 10.75 | 0.23 |
| Namibia | 71.62 | 0.30 | 14.54 | 0.06 | 57.08 | 0.24 |
| South Africa | 4535.13 | 0.58 | 673.54 | 0.09 | 3861.59 | 0.50 |
| Kingdom of Eswatini | 78.34 | 0.69 | 14.32 | 0.13 | 64.02 | 0.56 |
| Zimbabwe | 110.52 | 0.22 | 18.56 | 0.04 | 91.96 | 0.19 |
| Benin | 122.79 | 0.26 | 12.81 | 0.03 | 109.98 | 0.24 |
| Burkina Faso | 104.86 | 0.19 | 17.84 | 0.03 | 87.03 | 0.15 |
| Cameroon | 522.88 | 0.34 | 71.09 | 0.05 | 451.8 | 0.30 |
| Republic of Cabo Verde | 16.20 | 0.38 | 1.27 | 0.03 | 14.93 | 0.35 |
| Chad | 43.05 | 0.14 | 6.00 | 0.02 | 37.05 | 0.12 |
| Republic of C涔坱e d'Ivoire | 587.13 | 0.35 | 74.93 | 0.04 | 512.21 | 0.30 |
| Republic of the Gambia | 16.42 | 0.25 | 2.34 | 0.04 | 14.08 | 0.21 |
| Ghana | 869.46 | 0.38 | 92.94 | 0.04 | 776.52 | 0.34 |
| Guinea | 121.00 | 0.24 | 18.10 | 0.04 | 102.91 | 0.20 |
| Guinea-Bissau | 16.60 | 0.33 | 2.45 | 0.05 | 14.15 | 0.28 |
| Liberia | 17.42 | 0.21 | 2.02 | 0.02 | 15.41 | 0.18 |
| Mali | 153.09 | 0.27 | 14.12 | 0.02 | 138.98 | 0.25 |
| Mauritania | 44.70 | 0.17 | 7.15 | 0.03 | 37.56 | 0.15 |
| Niger | 42.72 | 0.11 | 5.57 | 0.01 | 37.15 | 0.09 |
| Nigeria | 2463.28 | 0.19 | 461.76 | 0.04 | 2001.52 | 0.16 |
| Sao Tome and Principe | 3.24 | 0.26 | 0.37 | 0.03 | 2.87 | 0.23 |
| Senegal | 198.53 | 0.30 | 23.9 | 0.04 | 174.63 | 0.26 |
| Sierra Leone | 55.39 | 0.22 | 7.07 | 0.03 | 48.32 | 0.19 |
| Togo | 35.8 | 0.17 | 5.94 | 0.03 | 29.86 | 0.14 |
| American Samoa | 3.69 | 1.06 | 0.10 | 0.03 | 3.59 | 1.03 |
| Bermuda | 35.88 | 0.61 | 6.08 | 0.10 | 29.80 | 0.51 |
| Greenland | 12.77 | 0.33 | 4.18 | 0.11 | 8.60 | 0.23 |
| Guam | 47.67 | 0.79 | 1.63 | 0.03 | 46.04 | 0.76 |
| Principality of Monaco | 47.66 | 0.59 | 9.88 | 0.12 | 37.77 | 0.47 |
| Republic of Nauru | 2.44 | 1.82 | 0.13 | 0.10 | 2.32 | 1.72 |
| Northern Mariana Islands | 8.05 | 0.79 | 0.26 | 0.03 | 7.79 | 0.77 |
| Republic of Palau | 4.36 | 1.54 | 0.18 | 0.07 | 4.17 | 1.47 |
| Puerto Rico | 1127.89 | 0.84 | 273.65 | 0.2 | 854.23 | 0.64 |
| Saint Kitts and Nevis | 11.97 | 0.76 | 1.81 | 0.11 | 10.16 | 0.64 |
| Republic of San Marino | 6.53 | 0.31 | 1.40 | 0.07 | 5.14 | 0.24 |
| Tuvalu | 0.43 | 0.64 | 0.03 | 0.04 | 0.40 | 0.60 |
| United States Virgin Islands | 40.96 | 1.02 | 8.87 | 0.22 | 32.1 | 0.80 |
| South Sudan | 7.87 | 0.11 | 2.02 | 0.03 | 5.86 | 0.08 |
| Sudan | 243.63 | 0.17 | 45.21 | 0.03 | 198.42 | 0.14 |
